# Bayesian Approach to Model CD137 Signaling in Human *M. tuberculosis* In Vitro Responses

**DOI:** 10.1371/journal.pone.0055987

**Published:** 2013-02-20

**Authors:** Darío A. Fernández Do Porto, Jerónimo Auzmendi, Delfina Peña, Verónica E. García, Luciano Moffatt

**Affiliations:** 1 Instituto de Química Física de los Materiales, Medio Ambiente y Energía, Facultad de Ciencias Exactas y Naturales, Universidad de Buenos Aires-CONICET, Buenos Aires, Argentina; 2 Instituto de Química Biológica - Ciencias Exactas y Naturales, -CONICET, Departamento de Química Biológica, Facultad de Ciencias Exactas y Naturales, Universidad de Buenos Aires, Buenos Aires, Argentina; University Medical Center Freiburg, Germany

## Abstract

Immune responses are qualitatively and quantitatively influenced by a complex network of receptor-ligand interactions. Among them, the CD137:CD137L pathway is known to modulate innate and adaptive human responses against *Mycobacterium tuberculosis*. However, the underlying mechanisms of this regulation remain unclear. In this work, we developed a Bayesian Computational Model (BCM) of *in vitro* CD137 signaling, devised to fit previously gathered experimental data. The BCM is fed with the data and the prior distribution of the model parameters and it returns their posterior distribution and the model evidence, which allows comparing alternative signaling mechanisms. The BCM uses a coupled system of non-linear differential equations to describe the dynamics of Antigen Presenting Cells, Natural Killer and T Cells together with the interpheron (IFN)-γ and tumor necrosis factor (TNF)-α levels in the media culture. Fast and complete mixing of the media is assumed. The prior distribution of the parameters that describe the dynamics of the immunological response was obtained from the literature and theoretical considerations Our BCM applies successively the Levenberg-Marquardt algorithm to find the maximum a posteriori likelihood (MAP); the Metropolis Markov Chain Monte Carlo method to approximate the posterior distribution of the parameters and Thermodynamic Integration to calculate the evidence of alternative hypothesis. Bayes factors provided decisive evidence favoring direct CD137 signaling on T cells. Moreover, the posterior distribution of the parameters that describe the CD137 signaling showed that the regulation of IFN-γ levels is based more on T cells survival than on direct induction. Furthermore, the mechanisms that account for the effect of CD137 signaling on TNF-α production were based on a decrease of TNF-α production by APC and, perhaps, on the increase in APC apoptosis. BCM proved to be a useful tool to gain insight on the mechanisms of CD137 signaling during human response against *Mycobacterium tuberculosis*.

## Introduction

Tuberculosis is one of the earliest recorded human diseases that still poses an unresolved global health problem. *Mycobacterium tuberculosis* (*M.tb*), the causative agent of tuberculosis, results in 2 million deaths annually worldwide despite available treatment. Furthermore, approximately one-third of the world population is estimated to be infected with *M.tb* (WHO, 2010).

Although the immunological mechanisms against *M.tb* are not fully understood, protective defense against mycobacterial infections is primarily mediated by the interaction of antigen-specific T cells and macrophages [1], [2]. This interaction often depends on the interplay of cytokines produced by these cells.

Even though a wide spectrum of cytokines may contribute to protection, a type 1 response, dominated by interferon (IFN)-γ secretion, is considered the main mediator of the protective immunity against *M.tb* [2], [3]. IFN-γ activates macrophages to become effector cells that express microbicidal substances and cytokines, with tumor necrosis factor α (TNF-α) playing a fundamental role in controlling the mycobacterial infection [4]–[5]. While the protective role of IFN-γ in tuberculosis is well established [2] [6], TNF-α exhibits a very complex network of interactions and many of its functions are still not fully understood [7]. In spite of the major role TNF-α plays a major role in controlling *M.tb* infection, activating macrophages early during the immune response and participating in granuloma formation [8], [9], excessive levels of TNF-α may cause tissue damage *in vivo*, including hyperinflammation and caseous necrosis [7].

Several signaling proteins modulate the levels and pattern of cytokines produced by immune cells upon *M.tb* antigen (Ag) stimulation [10]–[12]. In particular, we have demonstrated a key role of CD137 (4–1BB) in modulating human cytokine responses against *M.tb*. CD137 is a TNFR related superfamily signaling molecule that regulates the effector functions of most types of immune cells [13], [14]. We have previously shown that signaling through the CD137:CD137 ligand (L) pathway interfered with IFN-γ and TNF-α secretion by innate immune cells, while boosting T cell effector functions during tuberculosis [15]. However, these results did not provide a definite answer on the nature of the mechanisms of CD137 signaling. Therefore, we sought to develop a Bayesian Computational Model (BCM) in orfer to further our understanding of the mechanisms of this pathway. This BCM allowed us to fit previously gathered experimental data. By using the BCM we could predict the dynamics of Antigen Presenting Cells (APC), Natural Killer (NK) and T Cells together with the IFN-γ and TNF-α levels in the media culture. Bayes factors provided decisive evidence favoring direct CD137 signaling on T cells. Futhermore, the posterior distribution of the parameters that predicted that CD137 modulation on IFN-γ levels is based more on its effect on T cell survival than on direct induction of the cells. Besides, TNF-α regulation by CD137 was based on a reduction in TNF-α production by APC and, perhaps, on the increase in APC apoptosis. BCM proved to be a useful tool to gain insight on the mechanisms of CD137 signaling during human response against *Mycobacterium tuberculosis*.

Classical immunology uses conceptual models to make predictions and draw conclusions from experimental data, relying on the expert criteria of the researcher. Computational models arise from the formalization of those conceptual models and the expert criteria in a set of defined rules operating on simplified representations of the immunological process.

A BCM is meant to fit a set of actual experimental measurements. The probability function of the experimental observations is obtained using the error rate of the provided measurements. BCMs formalize the link between a qualitative hypothesis and experimental data. Our BCM applies successively: 1) the Euler method to solve the set of ordinary differential equations (ODE) that model the system to predict the outcome of the experiments, 2) the Square Sum of normalized residuals that compare predicted and measured values to calculate the posterior likelihood; 3) the Levenberg-Marquardt algorithm (LMA) to find the parameter that maximizes the posterior likelihood; 4) the Metropolis Markov Chain Monte Carlo method (MCMC) to sample from the posterior distribution of the parameters and 4) Thermodynamic Integration to calculate the evidence of alternative hypothesis about the signaling mechanism.

Here we present a parameterized BCM of a set of previous experiments performed to investigate the CD137 signaling pathway in tuberculosis and to gain an insight into the possible mechanisms of this pathway.

## Materials and Methods

### Experimental Data

The experimental rationale consists in studying the role of CD137 in the context of tuberculosis by using an anti-CD137 blocking monoclonal antibody (mAb) during proliferation, apoptosis and cytokine production. Most of the experimental data were from our previous work [15], with some additional kinetics results included here.

### Study Subjects

BCG vaccinated healthy adults (n = 40) with no history of tuberculosis participated in the study. Quantiferon TB Gold In-Tube® test (Cellestis INC, Valencia, CA, USA) was used to differentiate true healthy donors (HD) from individuals with latent tuberculosis, which were excluded from the study. HIV-negative patients (n = 40) with active tuberculosis (TB) were evaluated at the Hospital Muñiz (Buenos Aires, Argentina). The diagnosis of tuberculosis was established based on clinical and radiological data together with the identification of acid-fast bacilli in sputum. All participating patients had received <1 week of anti-tuberculosis therapy. Peripheral blood samples were collected in heparinized tubes from all individuals after receiving informed consent. The local ethical committee approved all the studies performed.

### Antigen

*In vitro* stimulation of cells throughout the present study was performed with a cell lysate from the virulent *M. tuberculosis* H37Rv strain (obtained through BEI Resources, NIAID, NIH: *Mycobacterium tuberculosis*, Strain H37Rv, Whole Cell Lysate, NR-14822) prepared by probe sonication. The antigen (Ag) preparation is indicated as “ *M. tb* Ag” throughout the manuscript.

### Culture Conditions

PBMC were isolated by density gradient centrifugation on Ficoll-Paque (Amersham Biosciences), resuspended in supplemented RPMI1640 and cultured (1×10^6^ cells/ml) in flat-bottom 24-welll or 96-well plates. In different experiments, cells were incubated in the presence/absence of *M.tb* Ag (10 µg/ml). At different times, CD137 and CD137L expression was determined by flow cytometry. For blocking experiments, cells were incubated 30 minutes with blocking mAbs (BD) against CD137, CD137L, or isotype control. Then, cells were stimulated with or without *M.tb* Ag. After 16 h, 4 or 5 days, the percentage of IFN-γ or TNF-α-secreting cells, lytic degranulation and apoptosis were determined by flow cytometry. For proliferation determination, cells were pulsed with [^3^H]TdR (1 µCi/well), harvested 16 h later and [^3^H]TdR incorporation was measured in a liquid scintillation counter. In separate experiments, mAbs anti-CD137 or anti-CD137L were added to cells with or without the specific Ag. After 16 h, 48 h or 5 days, IFN-γ and TNF-α production was evaluated by ELISA following the manufacturer’s instructions (eBioscience).

### Flow Cytometry

In different experiments, PBMC were cultured with *M.tb* Ag *±* CD137 or CD137L blocking mAbs and stained for CD3, CD4, CD8, CD56, CD14, CD137, CD137L expression using specific mAbs (BD). Intracellular cytokine staining was also performed to determine IFN-γ and TNF-α (eBioscience) production at the single-cell level as reported [16]. CD107a/b lysosome-associated membrane protein-1/2 expression was used to measure CD8^+^ T lymphocyte degranulation, as previously described [17]. In all cases, negative control samples were incubated with irrelevant, isotype-matched mAbs in parallel with the experimental samples. For apoptosis analysis, after 5 days of culture, the percentage of apoptotic/necrotic CD3^+^, CD3^+^CD4^+^ or CD3^+^CD8^+^ cells was determined using the Annexin V-FITC Apoptosis Detection Kit I (BD) following the instructions of the manufacturer.

### Bayesian Computational Model

The parameterized BCM was developed for the prediction of the previously described experiments. To build the BCM, we identified the relevant elements of the biological process that are needed to fit the experimental data based on the prior knowledge of the system. We included only the variables for which we have experimental data, excluding those cell types or cytokines for which we have not. The Bayesian approach implies that one must “forget” about the information present in the results we want to fit. In this way, the amount of information present in the experiments that was not present in the prior information can be quantified.

### Simulation of the Experimental Results and Calculation of their Likelihood

The BCM is fed with the experimental data with their standard errors and, for a given vector of parameters, it simulates the results and calculates the vector of the normalized residuals. Each normalized residual *ε_yi_* is obtained by taking the difference between the simulated and the experimental datum and dividing it by the experimental measurement error:
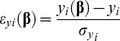
(1)where *y_i_*(**β**) indicates the predicted value of the *i^th^* observation as a function of the parameter vector **β**(explained below); *y_i_* indicates the actual measurement and *σ_yi_* indicates the standard error of the *i^th^* measurement. The parameter likelihood is evaluated via:
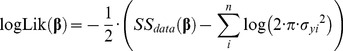
(2)where, SSdata, the squared sum of the residuals, is defined as



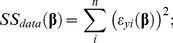
(3)The predicted values arose from the numeric approximation of a set of 17 nonlinear ordinary differential equations each one describing the time evolution of a particular variable of the idealized experimental system:
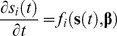
(4)where **β** is a vector comprising 77 parameters. The system variables include the number of cells in each state of the three modeled cells types and the levels of cytokines and Ag. The initial state of the system variables is also dependent on some components of the parameter vector:



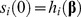
(5)This system of ODE equations (4) and the initial values of the state variables (5) are presented in the Supporting Information (Equations S1–S17 in Supporting Information S1). We took special care in choosing the variables and parameters so that each one represents an actual biological process. We only included one phenomenological variable, the proliferation ratio, which represents the cell capacity of the system.

Additional equations are used to relate the system variables with the expected value for each experimental data: the percentage of receptor/ligand expression for the included types of cells, the levels of IFN-γ and TNF-αin the media culture, the percentage of IFN-γ and or TNF-α-secreting cells, the apoptosis for T-cells and the rate of [^3^H]TdR incorporation by PBMC:
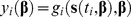
(6)


These equations are presented in Supporting Information S1 (R1–R14).

The ODE system of our BCM was constructed after the *M.tb*-immune system model developed by Marino [18]. We included NK cells and developed an approach for simulating co-stimulation and competition for receptor binding between the ligand and the anti-CD137 blocking mAb.

Simulations were performed for three different virtual treatments: first, the “*M.tb* treatment”; second, the “Blocking treatment” (*M.tb*+α-CD137) and finally, the “Media treatment” (control experiments).

From the point of view of plausible reasoning, the prior distribution of parameter values measures our knowledge (or ignorance) about the system. Therefore, we used experimental data obtained from different experimental contexts (including several models in vivo and in vitro) and/or theoretical considerations to determine the prior distribution of the BCM parameters (Table S1 in Supporting Information S1).

Parameters that indicate the scale magnitude of some property are better described after a logarithmic transformation, where equivalent uncertainties of scale are represented by adding or subtracting the same constant [19]. Alternatively, in the case of parameters describing ratios (the ratio of each type of cells in PBMC, the ratio of cells expressing the receptor, the ratio of cells producing cytokines), we chose a slightly different transformation to linearize uncertainty. We presumed that the mechanism responsible for ratio, *r*, could be modeled as a first order equilibrium constant, *r = E*/(*1+E*), hence *E = r*/(*1−r*). Therefore, for these parameters we applied a logit transformation, log(r)−log(1−r). In both cases, we describe our ignorance of the exact parameter values with a normal distribution of the logarithmic transformation of the parameters. The normal distribution is the probability density function that maximizes the entropy when only the mean and variance are known [19].

The solution of the ODE system was numerically approximated implementing the Euler method or the forth order Runge Kutta method in C++. Each one of the differential equations S1–S17 in Supporting Information S1 was approximated by assuming that all the state variables remained constant during each time step. Time steps of 6 seconds were used, since all the modeled biological processes occur in longer time scales. As those time scales were also longer that the mixing time of the system, we did not take into account local variations in the concentrations of the different components of the system. Instead, we approximated the evolution of the system as if there were an instantaneous and complete mixing of cells and cytokines. We modeled the evolution of a single average value for each state variable. This approximation, taking spatial structure out of the model, allowed for a considerable reduction in computational time and coding efforts.

Our model included three types of cell populations: Antigen Presenting cells (APC class), NK cells (NK class) and T cells (T class). We have previously demonstrated that CD137 and CD137L are both expressed on APC and NK cells, while only CD137 is expressed in lymphocytes after in-vitro *M.tb* stimulation [15]. To simplify the model, APC and NK are set to express the ligand and receptor simultaneously on the same cell, since it was reported that a receptor and ligand can be expressed on the same innate immune cell [20]–[21]. However, the implication of a single cell expressing both the ligand and the receptor and the immune outcome of bidirectional signaling by CD137/CD137L are not well understood [14]. Because the literature provides more evidence for reverse rather than direct signaling in monocytes, we only included the reverse signaling in these cells [22], [23]. On the other hand, we included only direct receptor signaling in NK and T cells, as there is no evidence for reverse signaling in these cell types. We modeled CD137 or CD137L signaling as a single instantaneous event.

CD137L stimulation in human monocytes has been shown to induce DC differentiation, with CD137L-DCs being more potent in stimulating T cell responses in vitro than the classical DCs. Taking into consideration these findings and the fact that we only had experimental data in CD14^+^ cells, we captured monocytes, macrophages and dendritic cells as a single APC population. Their importance lies in that macrophages are the preferred habitats of *M.tb* [24], and DCs are the major antigen presenting cells [25], [26].

During a persistent infection such as tuberculosis, CD137 and CD137L expression can be prolonged, therefore our model did not describe receptor internalization [14].

Because our focus was on the cytokine responses, we only included CD56^bright^ natural killer cells (NK^bright^) as they are the most efficient cytokine producers among NK populations [27]. Additionally, we excluded cytotoxic CD56^dim^ natural killer cells (NK^dim^) and CTL function in T cells. Finally, the entire T cell class (TL) captures both the CD4 and CD8 proinflammatory T cell subsets.

### APC Dynamics

**Figure 1:**
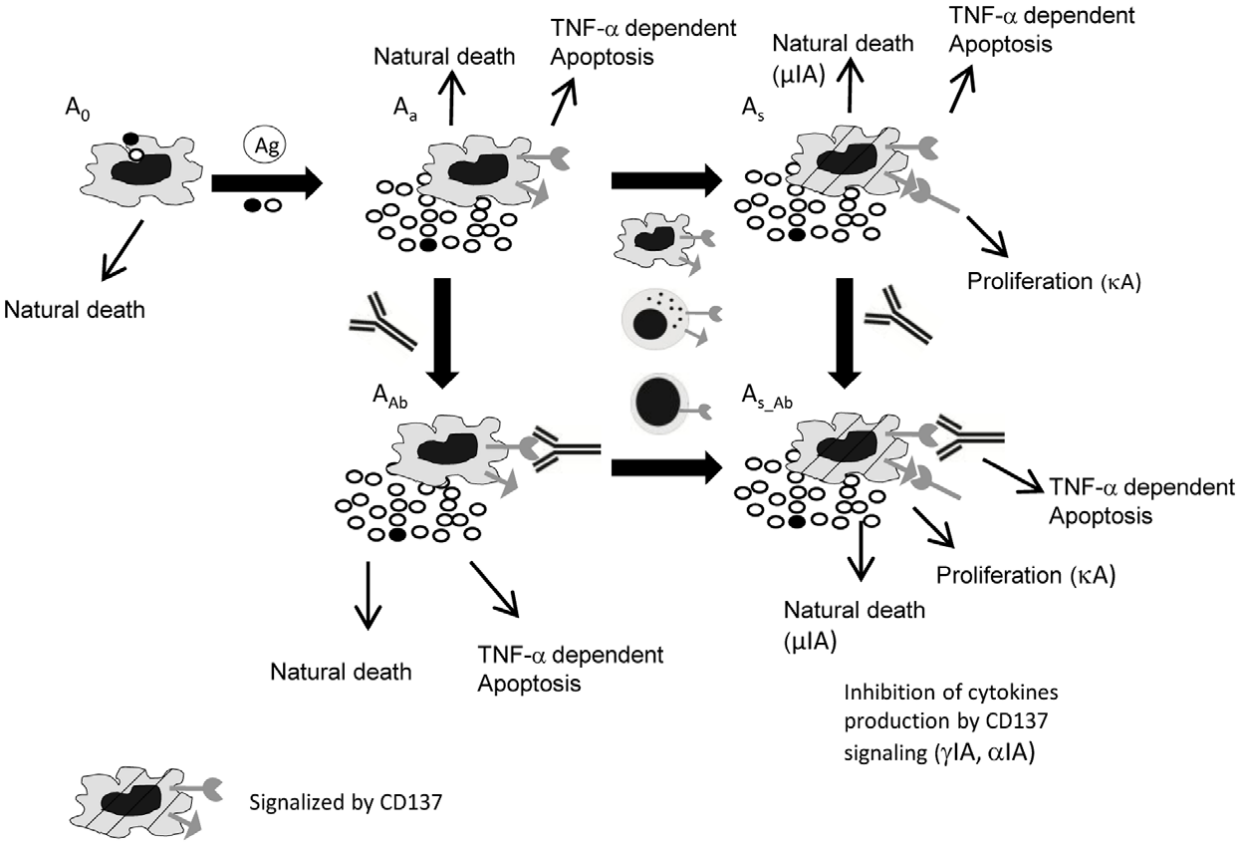
Diagram of APC dynamics in the in vitro culture. Five different APC subpopulations are described: resting (A_0_); activated (A_a_); activated and signaling through CD137 (A_s_); activated and bound to anti-CD137 blocking mAb (A_bl_); and activated, signaling through CD137L and bound to anti-CD137 blocking mAb (A_s_Ab_). Rows indicate possible mechanisms for each subpopulation. Loss of A_0_ is modeled with A_0_ uptake of the Ag (in the presence or absence of proinflammatory cytokines (macrophages and DC, respectively)) and death at a rate of µ_A0_. A small A_0_ ratio expresses receptor and ligand and produces basal levels of cytokines. A_a_ dynamics show the balance between APC uptake of Ag, natural death (µ_Aa_) and TNF-α-induced apoptosis (µα_A_). Once A_a_ interacts with other APC, NK or TL expressing CD137, signaling is initiated (A_s_). A_s_ dynamics includes proliferation, natural death and TNF-α induced apoptosis. A_Ab_ come from A_a_ receptor binding to blocking mAb. Although they have the receptor blocked, these cells can be reverse signalized by the antigen (A_s_Ab_). A_s_Ab_ also comes from A_s_ that bind the antigen. As we focus on CD137 signaling, parameters (cytokine production, proliferation and apoptosis rates) define two types of activated cells, determined by signaling through CD137 (A_s_,A_s_Ab_) or not (A_a_, A_Ab_). We assume that all activated APC express ligands and receptors and produce TNF-α, but only a fraction produces IFN-γ. The initial estimation for the induction of apoptosis, proliferation and cytokine secretion by CD137 can be either positive or negative.

We described five different APC subpopulations (Figure 1, Equations. S1–S5 in Supporting Information S1): resting (A_0_), activated (A_a_), activated and signalized through CD137 (A_s_), activated and bound to anti-CD137 blocking mAb (A_Ab_), and activated, signalized through CD137 and bound to anti-CD137 blocking mAb (A_s_Ab_).

A_0_ includes circulating undifferentiated monocytes and immature dendritic cells. Upon interaction with *M.tb*, dendritic cells undergo a number of phenotypical changes, a process termed maturation [26]. TNF-α and IFN-γ are also required for macrophage activation by the antigen [28]. Therefore, in our model the resting APC undergoes activation by the antigen uptake in a TNF-α and IFN-γ independent (DC, term K_(AxAg)_) or dependent (monocytes, term K_(AxAg)αγ_) manner. Thus, loss of A_0_ was modeled with the A_0_ antigen uptake and natural death at a rate of µ_A0_ (Equation (Eq.) S1 in Supporting Information S1). We defined natural cell death as all processes that end up in cell death with the exclusion of TNF-α dependent apoptosis. A small A_0_ fraction expresses receptor and ligand and produces basal levels of cytokines. This model included only classical macrophage activation.

Eq.S2 in Supporting Information S1 describes the A_a_ dynamics, showing a balance between APC Ag uptake (production of A_a_), natural death (µ_Aa_) and TNFα-induced apoptosis (µα_A_). Since we focused on CD137 signaling, the rate parameters describing cytokine production, proliferation and apoptosis were defined for two types of activated cells depending on whether they are signalized by CD137 (A_s_,A_s_Ab_) or not (A_a_, A_Ab_).

CD137 is expressed by primary monocytes in an activation dependent manner [29]. We assume that while all activated APC express ligand and receptor and produce TNF-α, only a fraction produce IFN-γ. There have been previous works showing that CD137 pathway induces activation, migration, survival, and differentiation on monocytic cells (monocytes, macrophages, and DCs) [23] [30]. However, it also seems likely that CD137 could play different roles depending on the infecting bacterial species. In fact, it has been proposed that CD137 plays opposite roles in Gram-negative and Gram-positive bacterial infections [31]. Therefore, in our model the initial guess for induction of apoptosis (IµA), and cytokine secretion (IαA IγA) by CD137 may be either positive or negative.

Preliminary results using cultures of purified monocytes stimulated with lysate of *M.tb* Ag, suggested that CD137 interacts with CD137L, both expressed on APC, causing a decrease in TNF-α secretion. It was also demonstrated that APC and NK activate each other during human response against *M.tb* [32] Thus, our model allows the interaction of ligand APC with NK receptor. Eq.S3 in Supporting Information S1 describes A_s_ dynamics. Once A_a_ population interacts with other APC or NK cells expressing CD137, it becomes signalized be CD137 (A_s_). If A_a_ presents the antigen to a naïve T cell, it can also become signalized via CD137 as long as the interaction is not blocked by an antibody. Traditionally, it was assumed that monocytes are unable to proliferate; however, it has been shown that CD137 induces a widespread proliferation of human peripheral monocytes [21]. Hence, we only allowed A_s_ to proliferate. The dynamics of these cells also include natural death as well as TNF-α-induced apoptosis.

Eq.S4 in Supporting Information S1 refers to A_Ab_ dynamics. These cells come from CD137 receptor in A_a_ binding to the blocking mAb. As they are involved in signaling, other term parameters are the same as in A_a_. Despite the receptor blockage, these cells can be modulated by reverse signaling through the antigen (A_s_Ab_). A_s_Ab_ also comes from A_s_ that binds the anti-CD137 blocking mAb (Eq. S5 in Supporting Information S1). These populations use the same term parameters as A_s._

### NK Dynamics

NK cell activity is regulated by a balance between the activating and inhibitory receptors [33]–[34]. Early studies demonstrated that mouse NK cell stimulation with cross-linking anti-CD137 antibodies or with CD137L-expressing cells induced NK cell proliferation and IFN-γ secretion [35]. However, it was recently demonstrated that CD137 is expressed by activated human NK cells and that this interaction reduced NK-cell activation and IFN-γ production. Additionally, it was shown that impaired NK-cell reactivity after CD137 triggering was not due to survival but rather to inhibitory signals [36].

Two major subsets of NK cells have been recognized in peripheral blood based on the differential expression of CD56 receptor [27]. The vast majority of circulating NK cells (CD56^dim^) are cytotoxic and do not produce IFN-γ. Only 5–10% of NK cells are IFN-γ producing cells (CD56^bright^). We have previously shown that CD137 and CD137L are expressed only on CD56^bright^ NK cells [15]. Considering our analysis was focused on cytokine modulation by CD137, we therefore included only the CD56^bright^ NK cells in our model.

**Figure 2:**
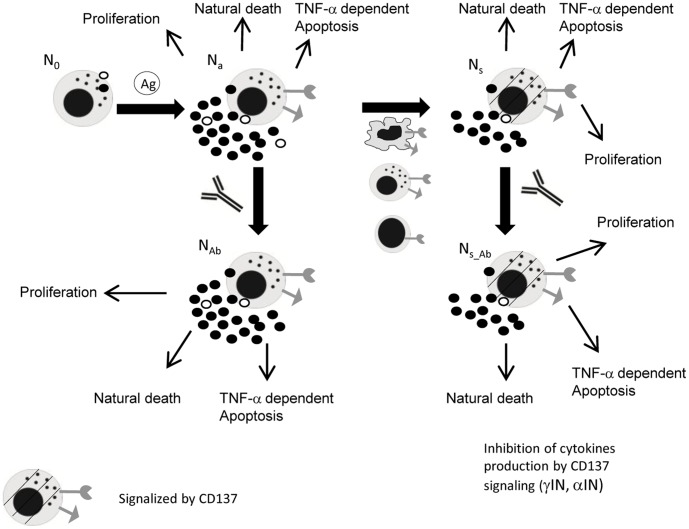
Diagram of CD56^bright^ NK cell dynamics in the in vitro culture. Five different NK subpopulations are described: resting (N_0_), activated (N_a_), activated and signaling through CD137 (N_s_), activated, signaling and blocked by anti-CD137 mAb (N_s_Ab_) and activated and blocked (N_Ab_). Rows indicate possible mechanisms for each subpopulation. The loss of N_0_ is modeled as NK activation (rate k_(N0,Na)A_) and death (µN_0_). N_0_ activation (N_a_) requires IL12 (indirectly modeled as activated APC), activated APC and *M.tb*. The model includes activation and signaling in two steps. Therefore, N_a_ dynamics includes CD137 signaling by APC or NK cells, natural death, TNF-α induced-apoptosis and proliferation. N_s_ dynamics includes IFN-γ/TNFα induction by CD137 (IγN, IαN). N_Ab_ is defined by equations similar to N_a_, but with the receptor bound to anti-CD137 blocking mAb. N_s_Ab_ behaves as N_s_, but the receptor is also blocked. We assume that all activated NK cells produce IFN-γ, but only a fraction produce TNF-α and expresse ligand/receptor pair.

Similar to APC dynamics, the model includes five NK cell stages (Figure 2, Equations S6–S10 in Supporting Information S1): resting (N_0_), activated (N_a_), activated with signaling though CD137 (N_s_), activated and blocked (N_Ab_) and activated with signaling and blocked by anti-CD137 mAb (N_s_Ab_).

CD56^bright^ NK cell activation in tuberculosis requires IL12, NK cell–APC interaction and *M.tb*–NK direct contact [37]–[38]. IL-12 is indirectly modeled through the presence of activated APC. As shown in Eq. S6 in Supporting Information S1, the loss of N_0_ is modeled as NK activation (rate k_(N0,Na)A_) and death (µN_0_). During NK activation, NK-CD137:CD137L-APC interaction is possible, but there is no actual evidence for this interaction. Thus, for the sake of clarity and simplicity, we chose to include activation and signaling in two separate steps. Additionally, because it was demonstrated that the NK-NK interaction is possible and that NK express both a ligand and a receptor, we incorporated this interaction as well. Hence, N_a_ dynamics (Eq. S7 in Supporting Information S1) include CD137 signaling by APC or NK cells, natural death, TNF-α induced-apoptosis and proliferation.

Eq. S8 in Supporting Information S1 describes N_s_ dynamics: this population consists of N_a_ signalized by the receptor. Eq. S9 in Supporting Information S1 describes N_Ab_, defined by the same parameters that N_a_, but with the receptor bound to anti-CD137 blocking mAb. Equation S10 in Supporting Information S1 describes N_s_Ab_ which behaves as N_s_, but with the receptor blocked. We assumed that all activated NK cells produced IFN-γ, but only a fraction produced TNF-α and expressed ligand/receptor pair.

### T Lymphocytes Dynamics

**Figure 3:**
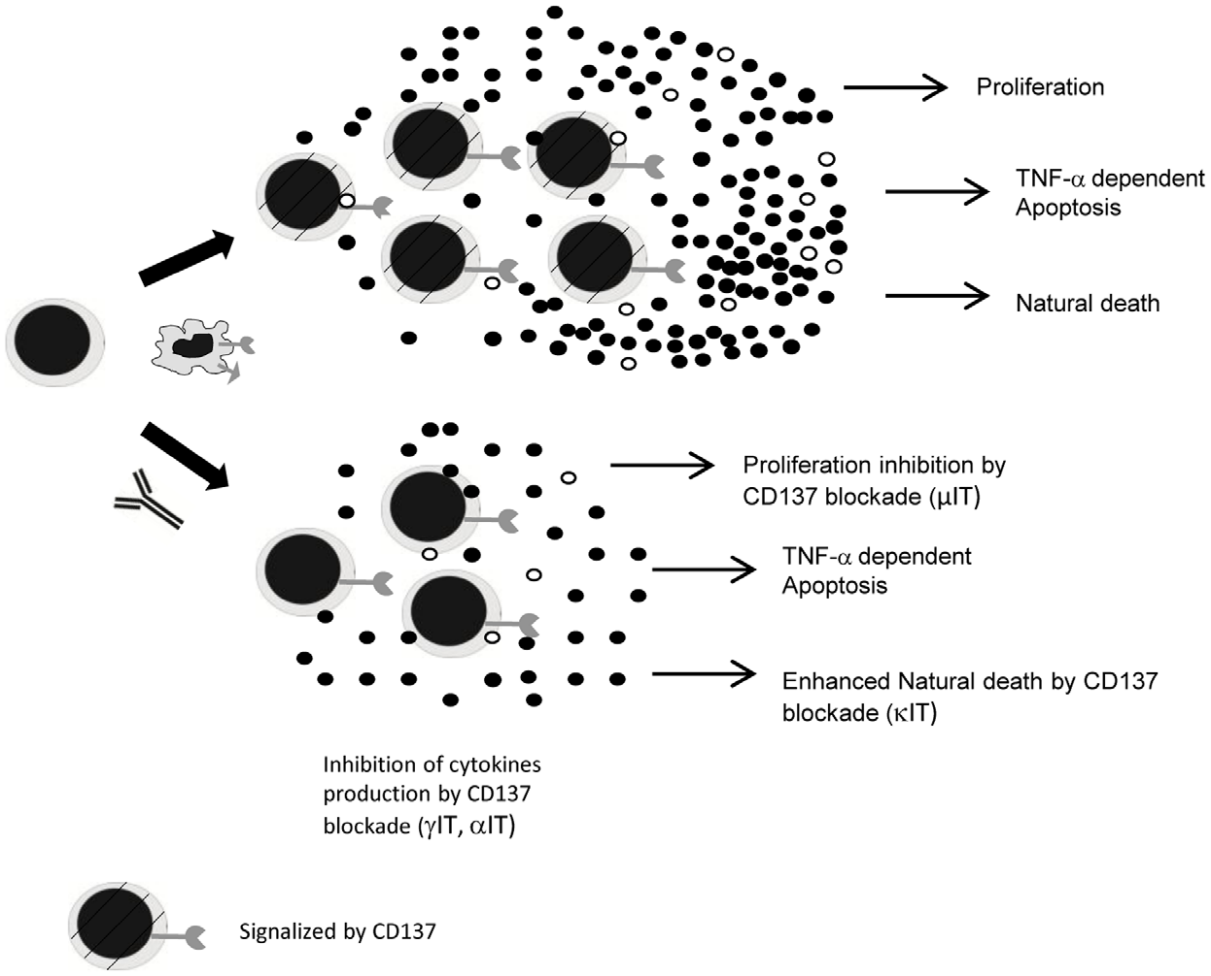
Diagram of TL dynamics in the in vitro culture. Four different TL subpopulations are described: non-specific-antigen-T cells (T_ns_), specific-antigen naïve T cells (T_0_), activated and CD137 co-stimulated cells (T_s_); and activated T cells with blocked CD137 (T_b_). Rows indicate possible mechanisms for each subpopulation. T_n_ population only proliferates and dies. T_0_ dynamics also includes proliferation and natural death at the same rate as T_n_, and can undergo activation/differentiation (and became T_s_ or T_b_) dependent on the presence of activated APC (A_a_, A_s_, A_s_Ab_ and A_Ab_). During activation, T cells express the receptor and, depending on the concentration of anti-CD137 mAb in the media, a portion of them can become blocked. Prior estimates indicate that CD137 signals delivered by agonistic antibodies or by overexpressed ligands can augment T-cell activation or survival. CD137 is not expressed on resting T cells, but rather is induced with antigen (Ag)-receptor signaling. T_s_ dynamics account for the differentiation of naïve LT_0_, proliferation, natural death and TNF-α induced apoptosis. T_bl_ dynamics incorporates apoptosis induced by CD137 blockage, inhibition and proliferation of IFN-γ and TNF-α production. The model assumes that all activated TL express the receptor.

T lymphocytes (TL) mediate adaptive immune responses that play a vital role in the elimination of *M.tb* [39]. We modeled four different T cells population: non-specific-antigen-T cells (T_ns_), specific-antigen naïve T cells (T_0_), activated and CD137 co-stimulated cells (T_s_) and activated T cells without CD137 signaling (because of mAb blockage) (T_bl_). (Fig. 3 Equations. S11–S14 in Supporting Information S1).

Non-specific T cells, T_ns,_ (Eq. S11 in Supporting Information S1), the more numerous component of PBMC *in vitro*, were included because their importance in the total cell count. Their population proliferates at a rate of κT_0_ and dies at a rate of µT_0_.

T_0_ dynamics are described in the equation S12 in Supporting Information S1. These cells proliferate and die at the same rate of T_ns_, but can undergo activation/differentiation that depends on the presence of activated APC (A_a_, A_s_, A_s_Ab_ and A_Ab_). The rate of activation was set to be independent from the CD137 receptor blockage state. During activation, T cells express the receptor that, depending on the concentration of anti-CD137 blocking mAb in the media, can become blocked.

Extensive evidence has shown that signals through CD137 delivered by agonistic antibodies or by an overexpressed ligand can augment T-cell activation or survival [40]–[43]. CD137 is not expressed on resting T cells, but is induced by antigen (Ag)-receptor signaling [41] [43]–[44]. Moreover, CD137 was proposed as an effector T cell marker [45]. Thus, we postulated that all activated TL express the receptor. Unpublished data from our lab demonstrated that only 43.82% ±1.63 and 23.95% ±3.85 of CD137^+^ TL are IFN-γ^+^ and TNF-α^+^_,_ respectively. Additionally, we proposed that CD137 blockade in T cells induced apoptosis and inhibited proliferation and cytokine production.

Equation S13 in Supporting Information S1 models T_s_ dynamics, accounting for the differentiation of naïve TL (T_0_), proliferation (with a rate of κT_s_), natural death (µT_s_) and TNF-induced apoptosis. Eq.S14 in Supporting Information S1 describes T_bl_ cell dynamics, incorporating CD137 blockage, induction of apoptosis, proliferation and IFN-γ and TNF-α production. We modeled apoptosis as occurring during time t_A_ (time duration of apoptosis); therefore, to predict the results of annexin binding, we counted the cells that entered apoptosis in a time window of t_A_ before the measurement took place (Eq. R10, Supporting Information S1).

### Culture Media Dynamics

Cytokines are produced by a large variety of cells involved in innate and adaptive immunity [46]. Because we measured IFN-γ and TNF-α levels in media and intracellular expression by single cells in our experimental data, we modeled these two key cytokines in tuberculosis.

Each equation has a degradation rate for each cytokine represented by a μ coefficient. TNF-α (Eq. S16 in Supporting Information S1) is mainly secreted by activated APC (at a rate of α-A_a_). Because CD137 might have opposite roles in TNF- α regulation during different bacterial infections [31]_,_ prior estimates for the induction of TNF-α (α-IA) by CD137 included both up (greater than one) and down (less than one) regulatory effects. The antigen presence enhances TNF-α production by A_a_. Additionally, TL and NK cells can secrete TNF- α; however, prior estimates indicated a low contribution from these cell types to the total TNF-α levels.

Human NK cells are known to be a major source of early IFN-γ (modeled in Eq. S15 in Supporting Information S1) upon *M.tb* stimulation in vitro [38]. In addition, prior estimates have indicated that CD137 inhibits cytokine production by NK cells [47]. On the other hand, macrophages were found to produce small levels of IFN-γ during *M.tb responses* [48]. Therefore, prior estimates indicated that activated APC produced a small amount of IFN-γ [49]. Once adaptive immunity has been fully developed, IFN-γ is mainly secreted by activated lymphocytes (γT_S_) [39]. CD137 enhances cytokine production by TL [50]–[51], thus, prior estimates for the receptor blockage were biased to values less than one.

When present, the antigen was modeled as being degraded with a rate constant of µAg (Eq S17 in Supporting Information S1). Tritiated Thymidine incorporation was calculated as the integral of all cell type proliferations that occurred for the last 16 h (Eq. R16). A scaling parameter *φTym* related this integral to the measured Thymidine incorporation in cpm.

### Posterior Likelihood Calculations

Prior probability of the tested parameters was calculated using normalized residuals analogous to the ones defined in Eq 1. The normalized residual corresponding to the j^th^ parameter, *ε_βj_*, was calculated by taking the difference between the log or logit transformed j^th^ parameter 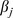 and the expected value of the log or logit transformed prior 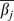 and dividing that value by the standard deviation 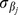
of the log or logit transformed prior:
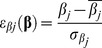
(7)


The square sum of the normalized parameter residuals definition is straightforward:
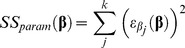
(8)


It was used to calculate the logarithm of the prior probability of the tested vector 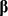:
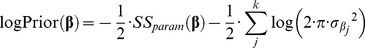
(9)


The logarithm of the posterior Likelihood function (logPostLik) results from taking the sum of the logPrior (Eq. 9) and the logLik (Eq. 2):
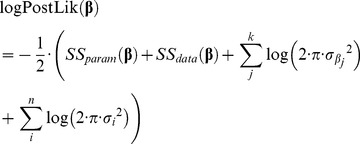
(10)


### Levenberg-Marquardt Algorithm and Maximum a Posteriori Likelihood

As only the square sums depend on the parameter vector, any parameter vector that locally minimizes the total squares sum (SS_tot_ = SS_param_+SS_data_) maximizes the *a posteriori* likelihood. Thus, we implemented in C++ a multivariate nonlinear least squares method, the Levenberg-Marquardt algorithm (LMA), to find MAP candidates. At each step of the algorithm, a new value of the transformed parameters’ vector, **β_new_**, was calculated
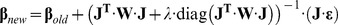
(11)where **J** and **W** are the Jacobian and weights matrices; **ε** is the residual vector and λ is the damping parameter. The elements of the Jacobian matrix are the partial derivatives of the *k* transformed parameters and of the n predictions measurements vector:



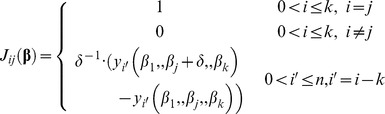
(12)A value of 10^−7^ was used for the incremental coefficient δ. Elements of the weight matrix, *W_ij_*, and elements of the residual vector, *ε_i_*, are defined using equations 1 and 7:
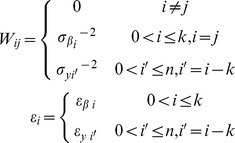
(13)


Initially, the damping parameter λ was set to 1000. At each LMA iteration (Eq. 11) the square sum for the new parameter vector was tested: when it diminished, λ was reduced by a factor of *v* (a value of 10 was used) and the new parameter vector was accepted; otherwise *v* was enlarged by the same factor and the old parameter vector was kept. The algorithm was run until either the change in Square Sum was less than 10^−9^ or 5000 iterations were reached.

At the vicinity of each local minimum, the gradient should be close to zero:
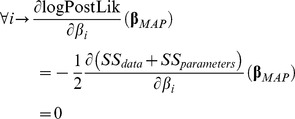
(14)


A small value in both λ and in the gradient (found by the matrix multiplication 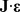) indicates that a local minimum and not a saddle point was found.

We chose different initial parameter vectors sampled from their prior distribution. Using the 2^nd^ order Taylor expansion we obtain an expression that approximates the logPostLik around its maximum.
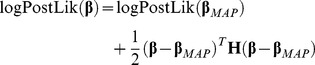
(15)where, **H** is the Hessian matrix, the second order derivatives of the logPosteriorLik:



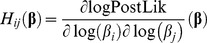
(16)Using eqs 10, 8, 7, 3 and 1, applying the chain rule, and neglecting the 2^nd^ order derivative we obtain the following approximation for the Hessian:
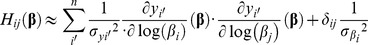
(17)where δ_ij_ equals one when i = j and zero otherwise. This Hessian approximation can be also expressed in terms of the Jacobian and weight matrices (eq 11 and 12):
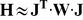
(18)The inverse of the Hessian matrix approximates the covariance of the posterior distribution of the parameters, 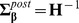.

The probability of finding the global MAP increases with the number of LMA runs.
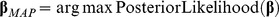
(19)


We performed 5000 different optimizations starting each one on a different initial value for the parameter to be fitted. The initial conditions were chosen using random samples from the prior distribution.

### Sampling the Posterior Distribution using Metropolis Monte Carlo Markov Chain

By using Metropolis Monte Carlo Markov Chain method we obtained a series of samples from the posterior likelihood, we then estimated the credible intervals (CI) of the parameters and by running simulations on the sampled parameters we obtained the predictive posterior intervals (PPI) of the simulated data. CI and PPI are analogous to the confidence intervals of frequentist statistics. For example, in an experiment that determines the uncertainty distribution of parameter p, if the probability that lies between ‘a’ and ‘b’ is 0.95, then a<p<b is a 0.95 CI or PPI. Metropolis MCMC method intends to sample from a target distribution by performing a random walk over the entire space of parameters. This random walk is governed by a Markov Chain where the ratio of the transition probabilities between points in the parameters space has to be equal to the ratio of the parameters’ probabilities. By setting proper transition probabilities we guarantee that, in the long run, we are sampling from the target distribution. Metropolis MCMC set the transition probability to be equal to the composition of an arbitrary symmetric jumping distribution and an acceptance probability that contains the actual information about the target distribution. However, the velocity of the convergence of the random walk is dependent on an appropriate selection of the jumping distribution.

We used a multivariate normal as the jumping distribution. The covariance of this distribution was set to be equal to the inverse of the Hessian approximation at the MAP multiplied by a constant factor. This factor was empirically adjusted so the acceptance ratio was roughly 20%. A factor of 0.02 was found to comply. To avoid excessive sample correlation we stored one every 231 MCMC steps (3 times the number of parameters).

### Calculating the Evidence and Bayes Factors

The use of Bayes factor for model comparison remains valid under less stringent conditions than other commonly used comparison tools like the likelihood ratio test (LRT), which rests on the assumption that the error in the parameter’s estimation follows a normal distribution. Bayes factors integrate prior information (which has to be stated on the prior distribution of the parameters) and place a penalty for placing too much model structure, in this way it guards against over-fitting. However, they are difficult to calculate, since marginalization involves integrating the posterior likelihood over the multidimensional space of parameters values. Bayesian model comparison depends on estimating the model Evidence, which is defined as the probability of obtaining the data given that the model is true and given the prior information available about the parameters of the model. Formally, we marginalize the parameters **β** of the model *M_i_*:
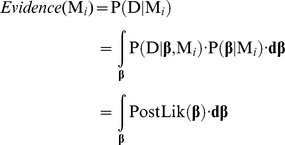
(20)


The model evidence can be used to compare the support the data gives to alternative models through the calculation of Bayes factors, which is the ratio of the evidences of the different models:
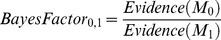
(21)


The value of the Evidence provides an absolute measure of the capability of the model to predict that data, irrespective of the number of parameters but depending on the prior information about them. To numerically approximate this integral we used a MCMC based method, Thermodynamic Integration [52], which builds a continuous and differentiable path of un-normalized distributions, *q_α_*, between the prior and the un-normalized posterior (the posterior likelihood):
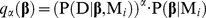
(22)


At α = 0, *q_α_* is the prior; at α = 1, *q_α_* is the posterior likelihood. The logarithm of the Evidence can be obtained by taking the expected value of the logLikelihood sampled along this path (see deduction in [52])
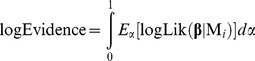
(23)where *E_α_* is the expectancy under *q_α_* distribution. This integral can be numerically solved by dividing the α path in N sections, α = (1/N, 2/N,… N/N) and running a Metropolis MCMC for the *q_αj_* distribution (eq. 22) at each *αj* value. Each MCMC run started at the last sample of the previous run. MCMC settings were the same as above except for the covariance of the jumping distribution. We used the product of the same constant factor (0.05) and the inverse of the Hessian approximation of the corresponding *q_α,_*



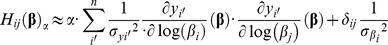
(24)At α = 1, Eq 18 reduces to Eq. 17.

## Results

### BCM Adequately Fits All Experimental Data

**Figure 4:**
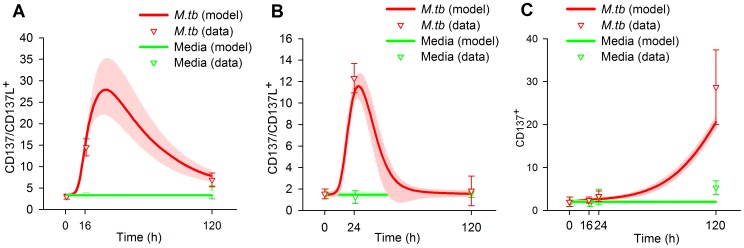
Fitting of the model (CD137/CD137L expression) to the data. A, Expression of CD137/CD137L in APC. B, Expression of CD137/CD137L on NK. C, Expression of CD137 on TL. Curves represent the best fit of our mathematical model to the data. The median and the 50% of the predictive posterior interval are shown. Means of experimental data are shown by triangles, error bars indicate the SEM from each group (7 individuals). Experimental data were obtained from PBMC of tuberculosis patients stimulated with *M.tb* Ag for 0, 16 and 120 h (A), 0, 24 and 120 h (B) or 0, 16, 24 and 120 h (C). CD137 expression was determined by flow cytometry. The cytometric analysis was performed by first gating on monocytes by light scatter and then gating on CD14^+^ cells (A), or by first gating on lymphocytes by light scatter and then on CD3^−^CD56^bright^ for NK cells (B) or on CD3^+^ for T lymphocytes (C). Predictions were made according to the following equations (Supporting Information S1): A: R4, B: R8, C: R10.

**Figure 5:**
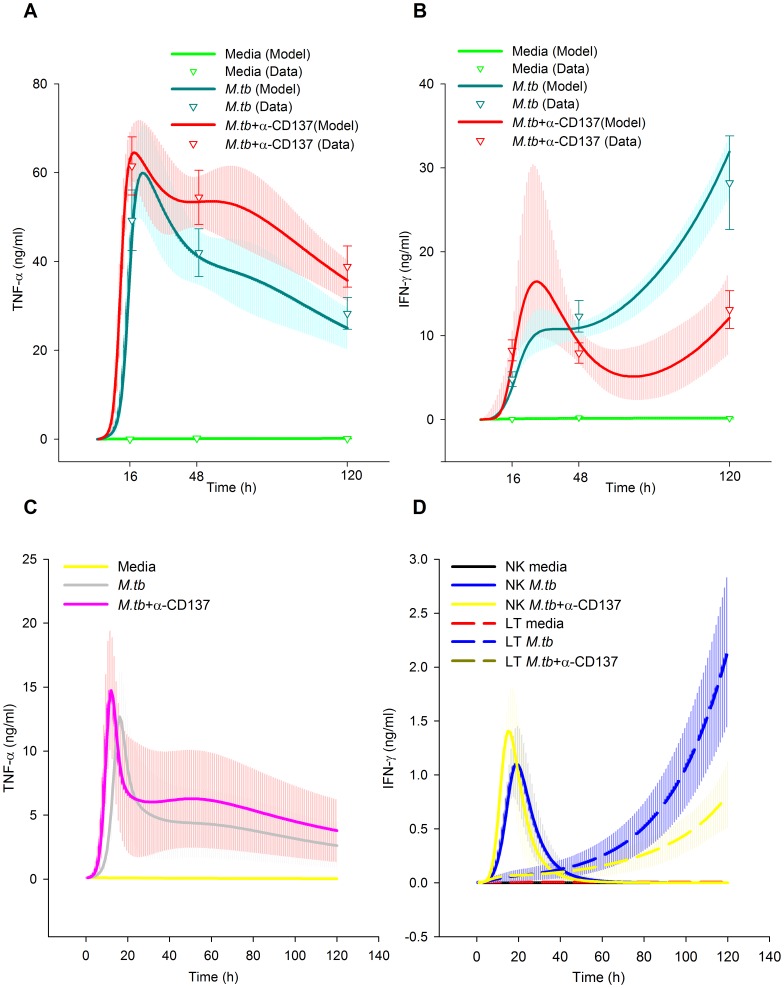
Role of CD137:CD137L pathway in the cytokine microenvironment during human tuberculosis. A,B; Fitting of the data model (cytokines in media). Curves represent the best fit of our mathematical model to the data. The median and the 50% of the predictive posterior interval are shown. Means of experimental data are shown by triangles ± SEM from each group (7 individuals). Experimental data were obtained from PBMC of tuberculosis patients stimulated with or without *M.tb* Ag in the presence or absence of CD137 blocking mAb. After 16 h (ON), 2 days or 5 days, cell-free supernatants were collected and assayed for TNF-α (A) and IFN-γ (B) production by ELISA. The mean ± SEM (15 individuals) of IFN-γ and TNF-α secretion levels is shown for each time. C, Predicted kinetic profile of instantaneous TNF-α production rate by total APC according to the BCM; the median and the 50% of the predictive posterior interval are shown. D, Simulation kinetics profile of instantaneous IFN-γ production rate by total NK and total TL; the median and the 50% of the predictive posterior interval are shown. Predictions were made according to the following equations (Supporting Information S1): A: S16 in Supporting Information S1, B: S15 in Supporting Information S1, C: S15′ in Supporting Information S1, D: S16′′ in Supporting Information S1 and S16′′′ in Supporting Information S1. New experimental data was included to validate the model. Levels of TNF-α and IFN-γ produced by PBMC stimulated with *M.tb* ± α-CD137 for 16 and 24 hours were measured by ELISA. IFN- and TNF-α levels of the new data was normalized as following: Normalized data=new data old mean of M.tb treatment at 16 h/new mean of M. tb treatment at 16 h. Normalized data is shown by bold triangles.

**Figure 6:**
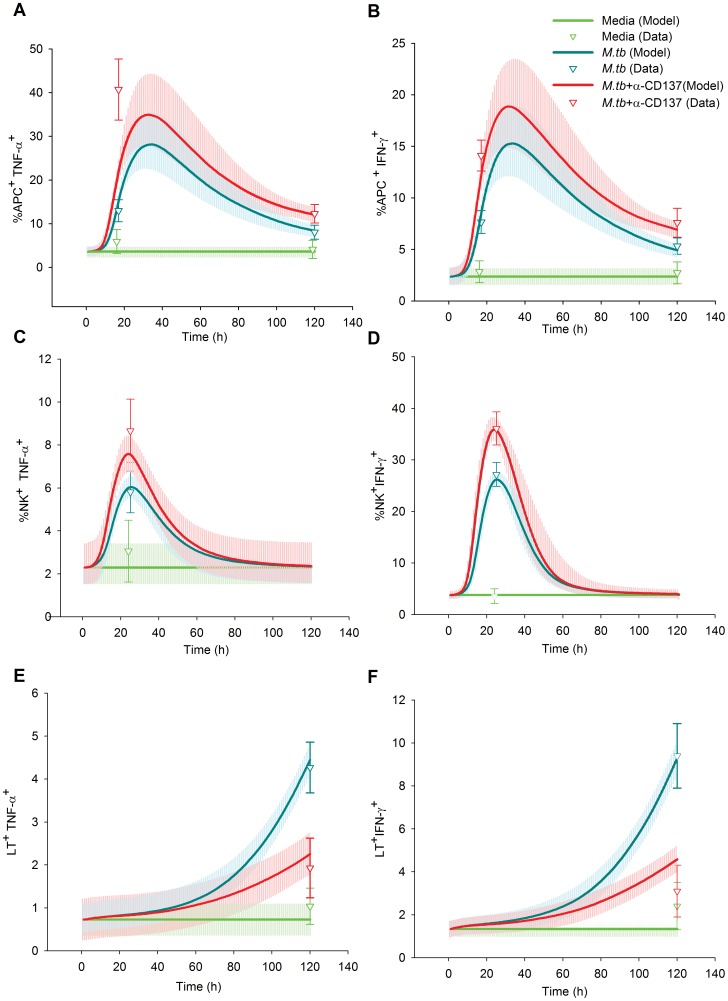
Effect of CD137:CD137L pathway on the immune cell cytokine production in tuberculosis. Different curves represent the best fit of our mathematical model to the data, the median and the 50% of the predictive posterior interval are shown. The mean of the experimental data is shown by triangles. Experimental data were obtained from PBMC of tuberculosis patients stimulated with *M.tb* Ag in the presence or absence of blocking anti-CD137 mAb. A–B; After 16 and 120 h, the intracellular expression of TNF-α (A) and IFN-γ (B) was determined by flow cytometry, by first gating on monocytes by light scatterand then by gating on CD14^+^ cells. Each represents the mean ± SEM of the percentage of CD14^+^cytokine^+^ cells for each group (11 individuals). C–D; PBMC were stimulated with *M.tb* Ag for 24 h in the presence or absence of blocking anti-CD137 mAb and intracellular TNF-α (C) and IFN-γ (D) expression on CD56^bright^ NK cells was determined by flow cytometry by first gating on lymphocytes by light scatter, then by gating on CD3^−^ cells and finally gating on CD56^bright^ NK cells. Each triangle represents the mean ± SEM (10 individuals). E–F; PBMC were stimulated with *M.tb* Ag for 4 days in the presence or absence of anti-CD137 blocking mAb. Intracellular TNF-α (E) and IFN-γ (F) expression was determined by flow cytometry in T cells. Each triangles represents the mean ± SEM (16 individuals). Predictions were made according to the following equations (Supporting Information S1): A: R3, B: R2, C: R7, D: R6, E: R12, F: R11.

**Figure 7:**
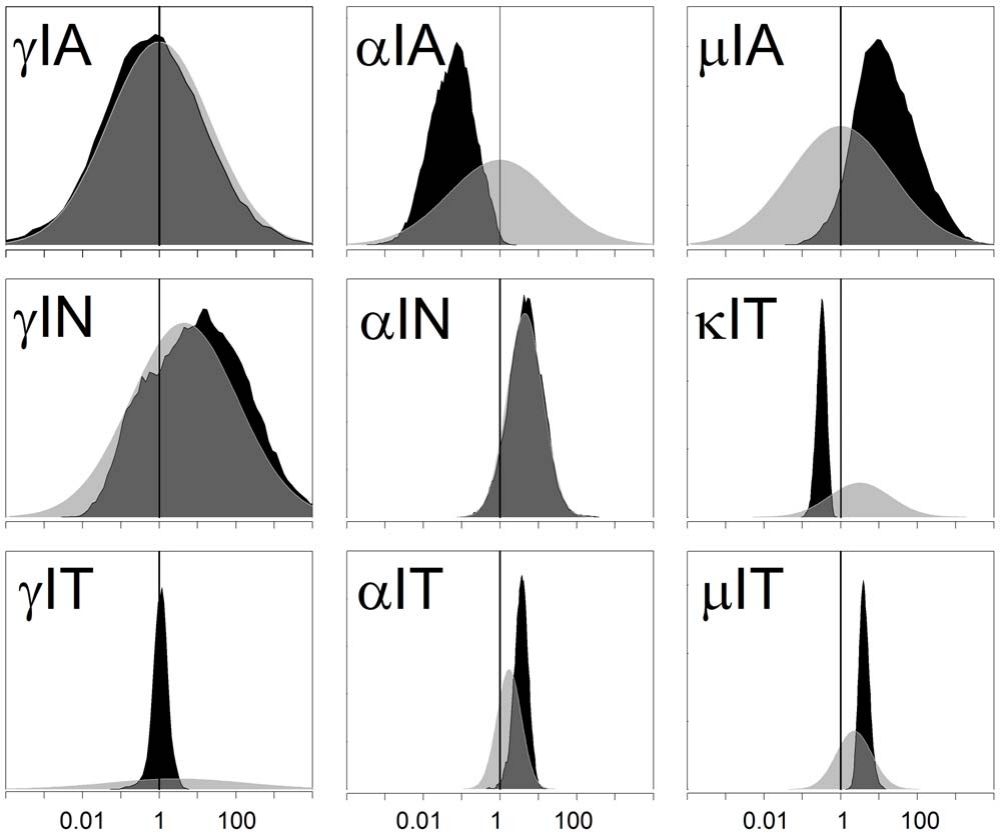
Reduction in the uncertainty of model parameters that describe the CD137 induction on cytokine production, proliferation and apoptosis rates. Bayesian analysis on the experimental data reduced the uncertainty of the BCM parameter values. Light gray areas represent the prior parameter distribution. Black areas represent the posterior parameter distribution. γIA (Induction factor of IFN-γ production by CD137::CD137L of APC), αIA (Induction factor of TNF-α production by CD137::CD137L of APC), µIA (Induction of death by CD137::CD137L of APC), γIN (Induction factor of IFN-γ production by CD137::CD137L of NK), αIN (Induction factor of TNF-α production by CD137::CD137L of NK), γIT (Induction of IFN-γ production by CD137), αIT (Induction of TNF-α production by CD137), µIT (Induction of apoptosis rate by CD137), κIT (Induction of proliferation rate by CD137).

We fed the BCM with an experimental series acquired during an already published study [53], with the addition of some unreported data. We also fed the BCM with the prior distribution of the parameters. Then, we took 5000 samples of the prior distribution and started a LMA from each one. Three times, after 287–807 iterations, LMA reached a value of 60.67 for SS_total_, the minimum value we reached, corresponding to SS_data_ 35.17 and SS_param_ 25.50, with a gradient norm of 0.02–0.002 and a λ value of 0.02 or lower. The difference in the parameters values was less than 1 in 3000, indicating that the same minimum was reached the three times. To check the validity of the Euler method we solved the ODE system with the forth order Runge Kutta method for this minimum. Both methods differed less than 0.36% in the prediction of data. We only use Euler method from this point on. Afterwards, we started 10 Metropolis MCMC runs at this minimum, taking 10000 samples of the posterior distribution of the model parameters at each run. The posterior distribution of the predicted data, which we will describe in the following sections, is shown in Figures 4, 5, 6 and the posterior distribution of the parameters in Figure 7 and Figures S1, S2, S3, S4, S5, S6, S7, S8, S9, S10.

We solved the ODE system with both the Euler method and the forth order Runge Kutta method for this minimum. We found that the predictions of the model using Runge Katta differed less than 0.36% from the predictions of the Euler method; therefore we decided to only use the latter for from this point.

### Expression Profile of CD137 in Innate and Adaptive Immune Cells

The timing of CD137:CD137L interactions and their effects on the immune cells may depend on the availability and induction of the ligand and receptor during the immune response [14]. Therefore, we studied the CD137 expression profile in the innate and adaptive immune cells during in vitro responses against tuberculosis. We measured the receptor expression in CD14^+^ monocytes, CD56^bright^ NK cells and CD3^+^ T cells using flow cytometry at different times after *M.tb* antigen stimulation. Minimal CD137 levels were measured on the surface of the three types of cells without stimulation. Overnight *M.tb* antigen stimulation induced CD137 expression on CD14^+^ cells (Figure 4A). Moreover, significant levels of CD137 were still detected on monocytes after 5 days. BCM simulation was able to fit this pattern and predicted a very distinctive profile. It predicted that CD137 takes 11 h to double its basal expression but only 4 h more to double it again. The expression was predicted to peak 30–35 h after the stimulus and gradually declined, taking 47 more hours to reach half the peak levels. After 120 h, the expression levels were still double the basal levels.

Flow cytometry also detected significant percentages of CD137 on CD56^bright^ NK cells at 24h of *M.tb* Ag stimulation, while only basal receptor levels were detected at 120 h. Data from the BCM simulation predicted that the peak occurred at 28 h, almost at the measurement time (Figure 4B). Simulations demonstrated an even longer delay than in APC, taking 17 h to double the basal levels and approximately 3 h to duplicate them again. In contrast, APC cells were predicted to return quickly to the basal levels, taking only 21 h to reduce the expression to two times the basal levels; fifteen hours later, the expression was only 10% higher than the basal levels, denoting an early role for these cells during in vitro immune response to *M.tb*.

The dynamics of CD137 expression was opposite in T lymphocytes (Figure 4C). Cytometry measurements displayed no significant increases in CD137 at 16 or 24 hours. However, after 120 h of stimulation with *M.tb* Ag, a 10-fold increase from the basal CD137 expression levels was measured in T lymphocytes. In the simulation, it takes `57 hours to double the basal levels, which thereafter continue augmenting until 120 h.

### Role of CD137 Pathway in Cytokine Modulation

Cytokines display a crucial role during the host immune response against *M.tb*. Thus, we investigated the role of CD137 pathway on cytokine modulation. These experimental results were previously published in [15]. Briefly, the results demonstrated that blocking CD137:CD137L pathway significantly augmented TNF-α production in tuberculosis patients at 16, 48 and 120 hours. BCM simulations could also successfully fit this pattern.

Predicted TNF-α levels in media during “*M.tb* treatment” (Figure 5A) present a delayed growth initiation followed by an exponential growth. Because the initial levels were set as zero, they could not be compared, so we compared the predicted cytokine levels at paired time points with or without *M.tb* stimulation (*M.tb* treatment and media treatment, respectively). After 9 hours of *M.tb* stimulation, the simulated TNF-α levels were doubled compared to the levels obtained with media treatment. Subsequently, TNF-α levels doubled 4 times during the following 9 hours. Predicted TNF-α levels reached a peak 22 hours after the stimulation. However, the levels diminished to approximately 50% of the maximal peak value by the end of the experiment.

Simulations predicted that blocking CD137 reduced the delay in TNF-α production. Accordingly, it predicted it only takes 3 h during the blocking treatment to double the TNF-α levels obtained with media treatment, doubling 5 more times in the 11 h thereafter. The levels peaked at 18 h, gradually diminishing thereafter to 50% of the peak value at the end of the experiment.

Additionally, we modeled IFN-γ production in PBMC. In line with our previous data, simulations demonstrated that blocking the CD137 pathway significantly augmented IFN-γ production after 16 h (ON) of *M.tb* stimulation (Figure 5B). However, the blockage of CD137 decreased IFN-γ secretion after 2 and 5 days of antigen stimulation (Figure 5B). With Ag treatment, IFN-γ levels were predicted to increase to double the levels without stimulus at 3 h.

Figure 5C prediction reveals that blocking CD137 results in accelerated an TNF-α production rate in APC, denoting a role of CD137 in changing the timing of the TNF-α response to *M.tb*. These curves share with those of TNF-α levels in media an initial exponential growth. Simulations demonstrated that neither NK nor TL produced significant levels of TNF-α. Figure 5D shows the NK and TL kinetics of the IFN- γ production rate. In line with the literature, our model predicts that IFN- γ is produced early by NK cells, with T cells acting as the major producers of this cytokine after the initial 26–35 h. APC were not observed to produce significant IFN-γ levels.

To validate the model, we measured TNF-α and IFN-γ production by PBMC stimulated with *M.tb* for 24 hours with or without anti-CD137 blocking antibodies, New experimental data presented no contradiction with the simulations (Fig. 5C).

### Intracellular Expression of Cytokines, Role of CD137

Our previous results [53] indicated that CD137:CD137L interactions might induce distinct effects on cytokine secretion in different cell types during the initial and later phases of the immune response. As shown in Figure 4A and 4B, CD137 blockade strikingly augmented the percentage of specific CD14^+^TNF-α^+^ and CD14^+^IFN-γ^+^ cells in ON responses to *M.tb*. Here, we provided previously unpublished results showing that CD137 blockade enhanced CD14^+^TNF-α^+^ and CD14^+^IFN-γ percentages at 5 days of *M.tb* treatment. Furthermore, BCM simulation once again fit the experimental results.

Additionally, CD137:CD137L pathway blockage augmented the number of CD3^−^CD56^bright^ TNF-α^+^ and CD3^−^CD56^bright^ IFN-γ^+^ NK cells in response to *M.tb* (Figure 6C and 6D). Simulation also predicted that the percentages of NK^+^TNF-α^+^ and NK^+^IFN-γ^+^ peak early during CD137 blockade.

Next, we investigated whether CD137:CD137L interactions regulated the percentage of IFN-γ and/or TNF-α producing lymphocytes. As shown in Figure 6E and 6F, CD137 blockade strikingly diminished the percentage of specific IFN-γ/TNF-α producing lymphocytes. Simulations predicted a slow exponential growth of the cytokine producing cells during the experimental time course.

### Additional Fitted Data

We included additional data related to the effect of CD137 blockage in our BCM analysis. *M.tb* Ag-stimulated PBMC proliferation ([^3^H]TdR incorporation) was decreased by CD137 blockage. Our BCM fit the measured [^3^H]TdR incorporation on all treatments. For media treatment the values compared were 2356–4675 cpm (0.25–0.75 confidence interval of the experimental data) vs. 3933–4914 cpm (0.25–0.75 posterior predictive interval obtained from simulations); for *M.tb* treatment: 12600–15700 cpm (experimental) vs. 11710–13331 cpm (simulations), blocking treatment [*M.tb*+anti-CD137 mAb]: 1414–9976cpm (experimental) vs. 374–11105 cpm (simulations). The effect of the blockage on the percentage of TL apoptosis (measured by cytometry) was also adequately fit: (media treatment: 11.3–21.8% CD3^+^ Annexin V^+^ [experimental] vs. 11.3–15.0% CD3^+^ Annexin V^+^ [simulations]; *M.tb* treatment*:* 24.3–30.9% CD3^+^ Annexin V^+^ [experimental] vs. 28.3–31.5% CD3^+^ Annexin V^+^ [simulations]; blocking treatment: 38.2–48.1% CD3^+^ Annexin V^+^ [experimental] vs. 36.7–42.1% CD3^+^ Annexin V^+^ [simulations]). A rough estimate of the cell number (5.00E5–1.50E6 cells for all times and treatments,) was also included in the BCM: (simulated data in media treatment: 24 h 7.30E5–8.85E6cells; 120 h: 7.38E5–9.82E6 cells; in *M.tb* treatment: 24 h: 7.28E5–8.81E6 cells 120 h: 8.85E5–1.17E6 cells; in blocking treatment 24 h; 7.25E5–8.78E6 cells 120 h; 7.88E5–1.04E6 cells).

### Reduction in Model Parameters Uncertainty

BCM analysis of the experimental data reduced the uncertainty in the model parameters’ values (Figures 7, S1, S2, S3, S4, S5, S6, S7, S8, S9, S10). The total prior distribution volume (measured as the logarithm of the covariance matrix determinant) was 16.4 dB. The posterior distribution volume was reduced to −962 dB, an average reduction of 12.5 dB per parameter. The average reduction in the variance of each parameter was of 6.91 dB, the difference between both reductions indicating a sizeable correlation between the parameters. Therefore, on average, the logarithmic range of each parameter was reduced by a factor 2.21 (3.46 dB). This reduction was not homogeneous: no changes occurred in 7 parameters, negligible reduction (less than 1 dB) in 21 parameters, moderate reduction (between 1 and 5 dB) occurred in 31 parameters and, finally, substantial reduction (between 5 and 14 dB) was observed in 22 parameters.

A reduction in the parameter distribution range indicates that the BCM successfully extracted from the data information that was not present in the prior. For certain parameters, it would be possible to extract the experimental information without this sophisticated model. For instance, the parameter rRLN_0_ (which suffered a reduction of 10 dB in its uncertainty) can be directly obtained from the Receptor/Ligand measurement in resting NK cells; a similar procedure could be applied to all ratios of resting cells. However, in most other cases (k(AxN), αIA_a_, κIT), the parameter depends on the data in a very complicated manner, requiring the application of BCM (or an equivalent approach) to extract the values.

An absence of range reduction is indicative of a poorly defined parameter, which is not surprising given that there are more parameters than data. A negligible reduction indicates that the experimental data contained little information about the parameter range. In certain cases (e.g., αN_0_, αN_a,_ γA_a_), the data contained no information about the parameters because the parameter influence on the data was negligible or because the effect on the data was confounded by other parameters. In other cases (e.g., γIN, rA, τAp), the parameter effect was sizeable but as there was substantial prior knowledge, the *new* data provided little additional information.

Those parameters that showed no reduction in the posterior range might be unidentifiable and they can be removed from the model without sizeable consequences. It is interesting to note that we were able to defer the responsibility of choosing the minimum set of parameters to the Bayesian Computational Model. We consider that this is an advantage of the BCM approach: in case of doubt one can incorporate a parameter to the model and still be able to predict the data and draw meaningful conclusions, although with the cost of a possibly quadratic increase in the computational time.

### Insight into the Response Dynamics and CD137 Mechanism

The effect of CD137 on cytokine production, proliferation and apoptosis rates is described by 9 parameters. Prior and posterior distributions of these parameters are shown in figure 7. In three cases (γIA,γIN and αIN) there is a close match between the prior and posterior distribution, indicating that the experimental series provides negligible information about the existence of these mechanisms. Using the Bayesian confidence interval method we calculated the probability that each one of the remaining parameters is greater or smaller than one (a value that indicates no CD137 effect). In the case of T cells, the regulation of IFN-γ production was found to be based more on the effect of CD137 on the survival of T cells (p(µIT<1)<0.00001 and p(κIT>1)<0.00001) than on IFN-γ induction (γIT). There was a huge reduction in the uncertainty of this last parameter, but with the posterior distribution centered on an induction value of one. On the other hand, the effect of CD137 on TNF-α levels appeared to be related to a direct inhibitory effect on TNF-α production by APC (p(µIA >1)<0.005 ) and, perhaps, to APC survival (p(µIA<1)∼0.06).

### Comparing Models using Thermodynamic Integration

**Figure 8:**
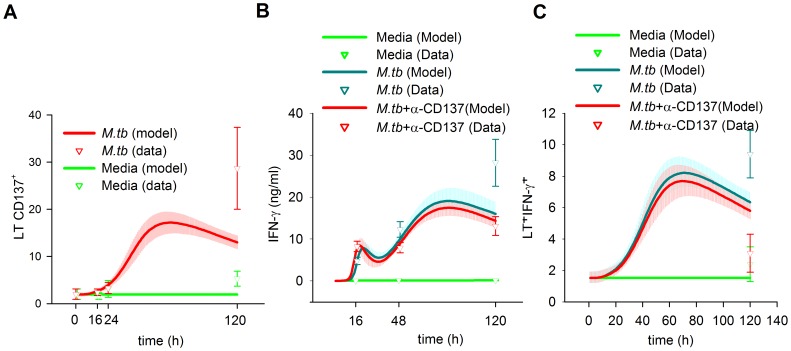
Predictions of the alternative model with indirect regulation over TL. A, CD137 expression on T cells; B, IFN-γ levels in media; C, TL intracelulIar IFN-γ expression. Curves represent the best fit of our mathematical model to the data. The median and the 50% of the predictive posterior interval are shown. Means of experimental data are shown by triangles ± SEM for each group (A, 7 individuals; B, 15 individuals; C, 16 individuals).

In our BCM, we modeled a direct CD137signaling effect in T cells. However, an alternative mechanism that also explains the late increase in IFN-γ through an indirect effect is possible. As blockage of the CD137/CD137L pathway increases TNF-α production by *in vitro* cultures of PBMC stimulated with *M.tb* Ag, it is possible that these higher TNF-α levels promote T cell apoptosis and, in consequence, diminish cell proliferation and cytokine secretion to a greater degree when compared to a direct CD137 blockade in T cells. To determine which of the competing mechanism is supported by the data, we compared the original model (“direct model”) with a new model where direct T cell signaling was absent (“indirect model”). This new BMC, of 73 parameters, was able to fit most of the experimental data, although not as precisely as the direct model, but it could not fit the CD137 blockade effects on IFN-γ production (Figure 8) and T cells survival. For this model, simulations predicted, for the percentages of T cells undergoing apoptosis, the following values: *Mt.b* treatment: 28.90–37.58%CD3^+^ Annexin V^+^, blocking treatment: 24.40–35.85%CD3^+^ Annexin V^+^. For. [^3^H]TdR incorporation: *Mt.b* treatment: 10855.7–15341.1 cpm, blocking treatment: 9527.9–14729.4 cpm.

The SS_total_ of the indirect model global minimum for 5000 starting points was 84.79, 24.12 units more than the SS_total_ of the direct model. We therefore calculated the Evidence of both models by Thermodynamic Integration. The evidence of the direct model was −284±1.68, while the indirect model evidence was −294.10±0.68. The difference between both evidences, 10.07, corresponded to a Bayes factor of 43.7 dB, which indicates decisive evidence in favor of the direct signaling of CD137 over T cells.

## Discussion

Mathematical models have been used to formulate hypotheses and theories and make predictions regarding both the immune response and *M.tb* infection that have previously posed a challenge with traditional experimental methods [54], [55]. In this paper, we set a different aim: to integrate the information contained in a particular experimental series with the previous knowledge by applying a Bayesian analysis. For this purpose, we developed a Bayesian Computational Model that simulated a set of previous experimental data to analyze the effect of CD137 signaling pathway during the human immune response against *M.tb* [15]. This BCM successfully fitted all the measured data and provided new information about many relevant biological parameters and complete kinetic profiles of the experimental variables. Moreover, the simulation results allowed us to postulate a mechanism responsible for the cytokine modulation by CD137.

BCM is made of two sets of equations: 1) a list of differential equations describing the initial state and the evolution of the underlying model, 2) a list of equations relating the state of the model with observable quantities. At the same time, BCM receives two kinds of inputs: 1) the prior distribution of parameters of those equations and 2) a set of experimental observations with their error rates. Finally, it produces three outputs: 1) the posterior distribution of the parameters, 2) probability distribution of the possible outcomes of observed and unobserved variables and 3) the evidence of the model.

FrequentistAs long as we avoid logical contradictions in the statement of the hypothesis and use proper priors, application of Bayes rule leads to valid conclusions irrespective of the sample size [19]There are many benefits provided by the BCM approach. First, it uses the prior knowledge present in the literature to interpret the experimental results analyzed. Also, it computes the information present in the results that was not present in the prior and, finally, it allows calculating the Bayes factors to assess the odds of alternative models. The resulting distribution of parameters can be used as priors for the analysis of new experimental results. In addition, the BCM allows incorporating new mechanisms: in this case, the data has to be re-analyzed under the new mechanism and one should run a new MCMC to obtain the evidence of the new model to compare it with alternative models. Finally, MCMC explores extensively the posterior distribution of the parameters for building the credible intervals of the parameters and the posterior predictive intervals of the data. In this way, spurious predictions that arise because of overfitting are averaged out. Methods like MAP or Maximum Likelihood, which are simpler and computationally cheaper, do not share this quality.

Other approaches share some but not all these possibilities. A classical ODE stability analysis might be complementary to the BCM approach as it would help to explain at least part of the reduction in range that happens in the distribution of the posterior parameters. However, this analysis is not designed to fit and extract information from experimental data. This task would be achieved by a classical non-linear least square fitting. Yet, this method does not incorporate prior information in a clear way, nor does it tolerate models with a smaller number of data than parameters. Finally, without the initial estimate of the covariance at or near the MAP, the MCMC method would not be able to approximate the posterior distribution in a feasible time. This estimate is a natural output of the MAP algorithm.

The system of ODE equations of the BMC is explicit and easy to solve by the Euler method. Euler method is known to be unstable when the time step duration is long enough for changes in the state variables to be significant in a single step. Given the nature of the simulated biological processes, we do not expect that to be the case for the chosen time step (6 seconds), and therefore expect the Euler method to be adequate. This expectation was corroborated by the small difference found for this time step between Euler and the fourth order Runge-Kutta methods.

LMA is a powerful technique that converges to a local minimum or a saddle point. Local minimum can be identified by having small values of both the gradient norm and the parameter λ. By starting at random points within the prior distribution of the parameters we allow the possibility of starting at the basin of attraction of the global minimum. By increasing the number of random starting points we increase the probability of finding the global minimum. Of 5000 random starts, 3 of them ended up with a Sum of Squares that was smaller than any other case, all the 3 with the same parameters values.

Our BCM is based on a large system of 17 coupled differential equations with 77 parameters. A large number of parameters and equations might be detrimental to the credibility of the model since the possibility of a spurious fit increases. Conversely, a small number of parameters and equations might not be enough to appropriately describe the system. In this paper, we used a small set of equations that describes the fundamental biological process necessary to explain the results. Every equation and the prior distribution of each parameter were based on the literature and theoretical considerations. The system of differential equations was built with the idea of reproducing the biological process that generates the data. Therefore, we did not avoid intricate dependencies between the APC, NK and T cells and cytokine levels that would make more difficult to fit the data. Moreover, as the values of parameters are constrained by their prior distribution, in principle there was no guarantee that a combination of parameters values would fit the experimental data. In fact just by fixing 4 parameters (the ones that describe CD137 direct signaling on T cells) the system fails to attain a complete fit of the data. Consequently, the sizeable number of equations and parameters compared to the analyzed experimental data was not an impediment for achieving valid results. Bayesian statistics is able to reach conclusions that are valid within its theoretical framework even in a suboptimal situation like the one we have dealt in this work. Orthodox statistics, on the other hand, cannot deal with small values of n. Such cases are simply passed over; since its focus is on large n. But small n is frequently the only information available. Fortunately, Bayesian analysis can deal with this situation: if we avoid logical contradictions in the statement of a problem and use proper priors, we can reach valid solutions [19]. Nevertheless, as the BCM is not based on the description of direct measurements of molecular interactions, the validity of the conclusions we reach about them are highly dependent on the validity of the model assumptions.

In our study we analyzed how CD137:CD137L interactions regulate cytokine secretion in different cell populations. Our previous experimental data demonstrated that CD137 blockage significantly augmented TNF-α production in PBMC at different times [15]. In line with those results, the posterior distribution of the parameters revealed that CD137 signaling in APC inhibited TNF-α production and enhanced apoptosis.

In agreement with the previous data, simulations demonstrated that blocking the CD137 pathway early significantly augmented IFN-γ production, while blocking this pathway late resulted in decreased cytokine secretion. We have previously hypothesized that CD137 signaling diminished IFN-γ production in NK cells at the early time points of *M.tb* Ag-stimulation and, later during the immune response augmented IFN-γ levels and T cell effector functions through the interaction between T cells and APC. Our simulations support this hypothesis. The posterior distribution of the parameters reveals that the mechanism for regulating TL cytokine production is based on the survival effect of the pathway more than on the inhibition of IFN-γ production. A simplified model that excluded direct TL could not fit the data on CD137 blockade effects on IFN-γ production and survival of T cells. Bayes Factor indicated decisive evidence for including the direct CD137 signaling in T cells. We therefore propose that the CD137 pathway regulates the mhomeostasis of cytokine levels required by the host to combat *M.tb* infection at different stages of the immune response.

Anti-CD137 antibodies are powerful immune modulators and have demonstrated promise in the therapeutic mouse models of cancer, viral disease, and autoimmunity. Moreover, phase I and II clinical trials using anti-CD137 therapy for advanced cancers are underway [56]. By performing Bayesian analysis of experimental data that included blocking mAbs, we obtained details about of the role CD137/CD137L pathway in regulating the immune response during active tuberculosis. We hope that this work will contribute to show how systems biology can be applied with experimental data in the design of pathways signaling and of therapeutics. Moreover, the results of our work may be used to design and evaluate animal knock out models of CD137 signaling and therapy. Nonetheless, caution must be used when designing ways to manipulate CD137 in human therapy, given that the agonistic anti-CD137 antibodies can cause severe immune system abnormalities [13], [56] and taking into consideration the fact that the CD137:CD137L pathway may operate differently in distinct cells during the innate and adaptive immune response.

## Supporting Information

Figure S1 **Reduction in the uncertainty of model parameters that describe proliferation rates of each cell.** Bayesian analysis on the experimental data reduced the uncertainty of the BCM parameter values. Gray areas represent ranges containing 50% of the prior parameter distribution. Black areas represent ranges containing 50% of the posterior parameter distribution. For a description of parameters see table S1 in Supporting Information S1.
(PDF)

Figure S2 **Reduction in the uncertainty of model parameters that describe death of each cell.** Bayesian analysis on the experimental data reduced the uncertainty of the BCM parameter values. Gray areas represent ranges containing 50% of the prior parameter distribution. Black areas represent ranges containing 50% of the posterior parameter distribution. For a description of parameters see table S1 in Supporting Information S1.
(PDF)

Figure S3 **Reduction in the uncertainty of model parameters that describe cytokine production rate by each cell.** Bayesian analysis on the experimental data reduced the uncertainty of the BCM parameter values. Gray areas represent ranges containing 50% of the prior parameter distribution. Black areas represent ranges containing 50% of the posterior parameter distribution. For a description of parameters see table S1 in Supporting Information S1.
(PDF)

Figure S4 **Reduction in the uncertainty of model parameters that describe ratios of cytokine producing cells.** Bayesian analysis on the experimental data reduced the uncertainty of the BCM parameter values. Gray areas represent ranges containing 50% of the prior parameter distribution. Black areas represent ranges containing 50% of the posterior parameter distribution. For a description of parameters see table S1 in Supporting Information S1.
(PDF)

Figure S5 **Reduction in the uncertainty of model parameters that describe ratios of receptor/ligand expressing cells.** Bayesian analysis on the experimental data reduced the uncertainty of the BCM parameter values. Gray areas represent ranges containing 50% of the prior parameter distribution. Black areas represent ranges containing 50% of the posterior parameter distribution. For a description of parameters see table S1 in Supporting Information S1.
(PDF)

Figure S6 **Reduction in the uncertainty of model parameters that describe ratios of cell populations.** Bayesian analysis on the experimental data reduced the uncertainty of the BCM parameter values. Gray areas represent ranges containing 50% of the prior parameter distribution. Black areas represent ranges containing 50% of the posterior parameter distribution. For a description of parameters see table S1 in Supporting Information S1.
(PDF)

Figure S7 **Reduction in the uncertainty of model parameters that describe antigen or antibody bindings.** Bayesian analysis on the experimental data reduced the uncertainty of the BCM parameter values. Gray areas represent ranges containing 50% of the prior parameter distribution. Black areas represent ranges containing 50% of the posterior parameter distribution. For a description of parameters see table S1 in Supporting Information S1.
(PDF)

Figure S8 **Reduction in the uncertainty of model parameters that describe saturation constants.** Bayesian analysis on the experimental data reduced the uncertainty of the BCM parameter values. Gray areas represent ranges containing 50% of the prior parameter distribution. Black areas represent ranges containing 50% of the posterior parameter distribution. For a description of parameters see table S1 in Supporting Information S1.
(PDF)

Figure S9 **Reduction in the uncertainty of model parameters that describe cell association.** Bayesian analysis on the experimental data reduced the uncertainty of the BCM parameter values. Gray areas represent ranges containing 50% of the prior parameter distribution. Black areas represent ranges containing 50% of the posterior parameter distribution. For a description of parameters see table S1 in Supporting Information S1.
(PDF)

Figure S10 **Reduction in the uncertainty of other model parameters.** Bayesian analysis on the experimental data reduced the uncertainty of the BCM parameter values. Gray areas represent ranges containing 50% of the prior parameter distribution. Black areas represent ranges containing 50% of the posterior parameter distribution. For a description of parameters see table S1 in Supporting Information S1.
(PDF)

Supporting Information S1 **Equations and table that describe the model and the model parameters.** Equations S1–S5, S6–10 and S10–S14 describe the dynamics of APC, NK and T cells respectively. Additional equations are used to relate the system variables with the expected value for each experimental data: the percentage of receptor/ligand expression for the included types of cells, the levels of IFN-γ and TNF-α in the media culture, the percentage of IFN-γ and or TNF-α-secreting cells, the apoptosis for T-cells and the rate of [^3^H]TdR incorporation by PBMC. Table S1 in Supporting Information S1 include a list of the parameter names, descriptions, units and prior and posterior parameters distribution. Parameters distributions are presented in ranges.
(DOCX)
