# Supplementary material for: Bayesian Approach to Model CD137 Signaling in Human M. tuberculosis In Vitro Responses
Source: PLoS One. 2013 Feb 20;8(2):e55987. doi: 10.1371/journal.pone.0055987 (PMC3577821; doi:10.1371/journal.pone.0055987)
Supplement: Figure S1 — Reduction in the uncertainty of model parameters that describe proliferation rates of each cell. Bayesian analysis on the experimental data reduced the uncertainty of the BCM parameter values. Gray areas represent ranges containing 50% of the prior parameter distribution. Black areas represent ranges containing 50% of the posterior parameter distribution. For a description of parameters see table S1 in Supporting Information S1. (PDF) [file pone.0055987.s001.pdf]

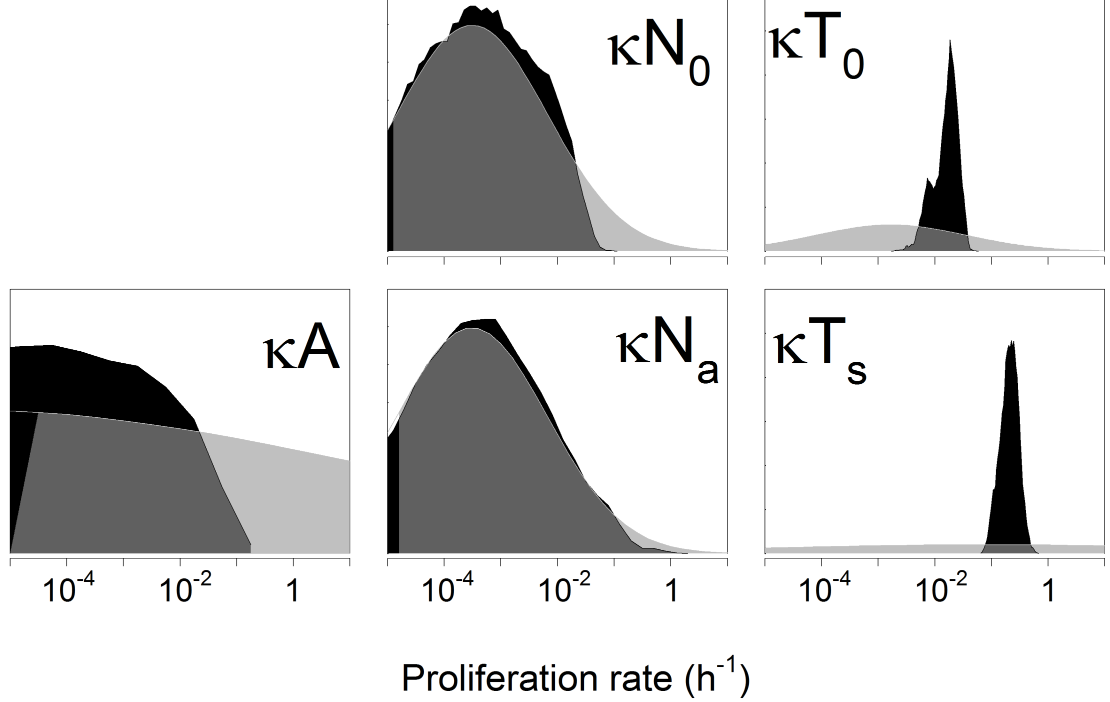

Figure S1. Reduction in the uncertainty of model parameters that describe proliferation rates of each cells. Bayesian analysis on the experimental data reduced the uncertainty of the BCM parameter values. Gray areas represent ranges containing 50% of the prior parameter distribution. Black areas represent ranges containing 50% of the posterior parameter distribution. For a description of parameters see table S1.
