# Supplementary material for: Bayesian Approach to Model CD137 Signaling in Human M. tuberculosis In Vitro Responses
Source: PLoS One. 2013 Feb 20;8(2):e55987. doi: 10.1371/journal.pone.0055987 (PMC3577821; doi:10.1371/journal.pone.0055987)
Supplement: Figure S10 — Reduction in the uncertainty of other model parameters. Bayesian analysis on the experimental data reduced the uncertainty of the BCM parameter values. Gray areas represent ranges containing 50% of the prior parameter distribution. Black areas represent ranges containing 50% of the posterior parameter distribution. For a description of parameters see table S1 in Supporting Information S1. (PDF) [file pone.0055987.s010.pdf]

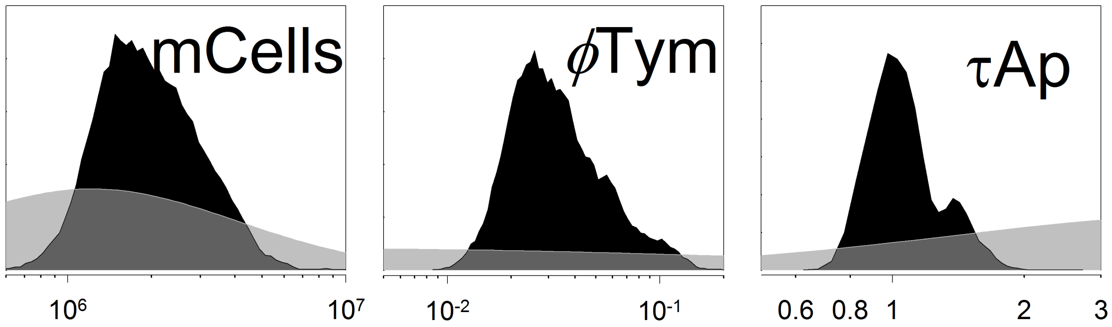

Figure S10. Reduction in the uncertainty of other model parameters. Bayesian analysis on the experimental data reduced the uncertainty of the BCM parameter values. Gray areas represent ranges containing 50% of the prior parameter distribution. Black areas represent ranges containing 50% of the posterior parameter distribution.
