# Supplementary material for: Bayesian Approach to Model CD137 Signaling in Human M. tuberculosis In Vitro Responses
Source: PLoS One. 2013 Feb 20;8(2):e55987. doi: 10.1371/journal.pone.0055987 (PMC3577821; doi:10.1371/journal.pone.0055987)
Supplement: Supporting Information S1 — Equations and table that describe the model and the model parameters. Equations S1–S5, S6–10 and S10–S14 describe the dynamics of APC, NK and T cells respectively. Additional equations are used to relate the system variables with the expected value for each experimental data: the percentage of receptor/ligand expression for the included types of cells, the levels of IFN-γ and TNF-α in the media culture, the percentage of IFN-γ and or TNF-α-secreting cells, the apoptosis for T-cells and the rate of [3H]TdR incorporation by PBMC. Table S1 in Supporting Information S1 include a list of the parameter names, descriptions, units and prior and posterior parameters distribution. Parameters distributions are presented in ranges. (DOCX) [file pone.0055987.s011.docx]

**SUPPORTING INFORMATION**

**Bayesian Approach to Model CD137 Signaling in Human *M. tuberculosis* in vitro Responses**

Darío A. Fernández Do Porto**^*^**, Jerónimo Auzmendi**^*^**, Delfina Peña**^†^**, Verónica E. García**^†^**, Luciano Moffatt**^*^**

**ODE equations**

**APC cells dynamics**

Resting (uncommitted) APC (A_0_)

A_0_ activation in inflammatory context (macrophages)

A_0_ activation (inflammatory context independent) (DC)

A_0_ death

$\frac{dA_{0}}{dt}=-\text{μA}_{0}\cdot A_{0}-\text{k}_{\left(\text{AxAg}\right)}\cdot Ag\cdot A_{0}-\text{k}_{\left(\text{AxAg}\right)\alpha\gamma}\cdot\left(\frac{\gamma}{\gamma+\text{K}_{\text{γ(A0,Aa)}}}\right)\cdot\left(\frac{\alpha}{\alpha+\text{K}_{\text{α(A0,Aa)}}}\right)\cdot Ag\cdot A_{0}$ (S1)

TNF-α induced apoptosis of APC

Activated APC (A_a_)

A_a_ death

$$A_{a}(0)=0$$

$\begin{matrix} \frac{dA_{a}}{dt}= \text{k}_{\text{(AxAg)}}\cdot Ag\cdot A_{0}\text{+k}_{\text{(AxAg)}\alpha\gamma}\cdot\left(\frac{\gamma}{\gamma+\text{K}_{\text{γ(A0,Aa)}}}\right)\cdot\left(\frac{\alpha}{\alpha+\text{K}_{\text{α}\left(\text{A0,Aa}\right)}}\right)\cdot Ag\cdot A_{0}-\text{μA}_{0}\cdot A_{a}-\text{μαA}\cdot\left(\frac{\alpha}{\alpha+\text{k}_{\alpha}\text{μA}}\right)\cdot A_{a} \\ -\text{k}_{\text{(AxA)}}\cdot\left(2A_{a}+A_{s}\right)\cdot A_{a}-\text{k}_{\text{(AxN)}}\cdot(\text{rRLN}_{\text{a}}\cdot N_{a}+N_{s})\cdot A_{a}-\text{k}_{\text{(AxT)}}\cdot\left(\frac{\text{k}_{\text{(TxAb)}}}{\text{k}_{\text{(TxAb)}}+Ab}\right) \cdot T_{0} \cdot A_{a}-\text{k}_{\text{(AxAb)}}\cdot Ab\cdot A_{a} \end{matrix}$ (S2)

A_a_ CD137 signaling by APC

A_a_ binding to

blocking anti-CD137 mAb

A_a_ CD137 signaling by NK

A_a_ CD137 signaling by TL

CD137:CD137L

induced proliferation of APC_a_

Activated APC with CD137L signaling(As)

$$A_{s}(0)=0$$

$\begin{matrix} \frac{dA_{s}}{dt}=\text{k}_{\text{(AxA)}} \cdot\left(2A_{a}+A_{s}\right)\cdot A_{a}+\text{k}_{\text{(AxN)}}\cdot\left(\text{rRLN}_{\text{a}}\cdot N_{a}+N_{s}\right)\cdot A_{a}+\text{k}_{\text{(AxT)}}\cdot\left(\frac{\text{k}_{\text{(TxAb)}}}{\text{k}_{\text{(TxAb)}}+Ab}\right) \cdot T_{0}\cdot A_{a} +\text{κΑ}\cdot r\kappa\cdot A_{s} \\ - \text{μ}_{\text{A}_{\text{a}}}\cdot\text{μIA}\cdot A_{s}-\text{μα}_{\text{A}}\cdot\left(\frac{\alpha}{\alpha+\text{K}_{\text{α}}\text{μA}}\right)\cdot A_{s}-\text{k}_{\text{(AxAb)}}\cdot Ab\cdot A_{s} \end{matrix}$ (S3)

CD137:CD137L

induced death of APC_a_

TNF-α induced

apoptosis of APC

A_s_ binding to

blocking anti-CD137 mAb (mAb values are constant)

Activated APC with the receptor bound to blocking anti-CD137 mAb (A_Ab_)

TNF-α induced apoptosis of APC

A_Ab_ death


$\begin{matrix} \frac{dA_{Ab}}{dt}= \text{k}_{\text{(AxAb)}}\cdot Ab\cdot A_{a}- \text{μ}_{\text{A}_{\text{a}}}\cdot A_{Ab}-\text{μα}_{\text{A}}\cdot\left(\frac{\alpha}{\alpha+\text{k}_{\text{α}}\text{μΑ}}\right)\cdot A_{Ab}- \text{k}_{\text{(AxA)}}\cdot\left(2A_{a}+A_{s}\right)\cdot A_{Ab}- \\ \text{k}_{\text{(AxN)}}\cdot A_{a}\cdot(\text{rRLN}_{\text{a}}\cdot N_{a}+N_{s})-\text{k}_{\text{(AxT)}}\cdot\left(\frac{\text{k}_{\text{(TxAb)}}}{\text{k}_{\text{(TxAb)}}+Ab}\right) \cdot T_{0}\cdot A_{Ab} \end{matrix}$ (S4)

A_Ab_ CD137 signaling by APC

A_Ab_ CD137 signaling by NK

A_Ab_ CD137 signaling by TL

Activated APC, signalized by CD137::CD137L and with the receptor bound to blocking anti-CD137 mAb (A_s_Ab_)

$$A_{s\_Ab}(0)=0$$

$\begin{matrix} \frac{dA_{s\_Ab}}{dt}=\text{k}_{\text{(AxAb)}}\cdot Ab\cdot A_{s}+\text{k}_{\text{(AxA)}}\cdot(A_{a}+A_{s})\cdot A_{Ab}+\text{k}_{\text{(AxN)}}\cdot(\text{rRLN}_{\text{a}}\cdot N_{a}+N_{s})\cdot A_{Ab}+ \\ \text{κ}_{\text{A}}\cdot r\kappa\cdot A_{s\_Ab}- \text{μ}_{\text{A}_{\text{a}}}\cdot\text{μIA}\cdot A_{s\_Ab}-\text{μαA}\cdot\left(\frac{\alpha}{\alpha+\text{K}_{\text{α}}\text{μA}}\right)\cdot A_{s\_Ab} \end{matrix}$ (S5)

TNF-α induced

apoptosis of APC

CD137:CD137L

induced death of APC_a_

CD137:CD137L

induced proliferation of APC_a_

**NK cells dynamics**

Resting (uncommitted) NK (N_0_)

NK_0_ proliferation

NK_0_ death

N_0_ activation induced by the antigen, APC and IL-12

(S6)

Activated NK (N_a_)

$$N_{a}(0)=0$$

N_a_ proliferation

$\begin{matrix} \frac{dN_{a}}{dt}=\text{κ}_{\text{Na}}\cdot r\kappa\cdot N_{a}+\text{k}_{\left(\text{N}0,\text{Na}\right)\text{A}}\cdot\left(\frac{A_{a}+ A_{Ab}+\text{αIA}\cdot(A_{s}+A_{s\_Ab})}{A_{a}+ A_{Ab}+\text{αIA}\cdot(A_{s}+A_{s\_Ab})+\text{K}_{\text{A}(\text{N}0,\text{Na})}}\right)\cdot(A_{a}+ A_{Ab}+A_{s}+A_{s\_Ab})\cdot Ag\cdot N_{0} \\ -\text{k}_{\text{(NxN)}}\cdot\text{rRLN}_{\text{a}}\cdot(\text{rRLN}_{\text{a}}\cdot2N_{a}+N_{Ab}+N_{s}+N_{s\_Ab})\cdot N_{a}-\text{k}_{\text{(AxN)}}\cdot\text{rRLN}_{\text{a}}\cdot(A_{a}+A_{s}+A_{Ab}+A_{s\_Ab})\cdot N_{a} \\ -\text{k}_{\text{(NxAb)}}\cdot N_{a}\cdot Ab-\text{μ}_{\text{Na}}\cdot N_{a}-\text{μαN}\cdot\left(\frac{\alpha}{\alpha+\text{K}_{\text{A}(\text{N}0,\text{Na})}}\right)\cdot N_{a} \end{matrix}$(S7)

N_a_ CD137L signaling by NK

N_a_ CD137L signaling by APC

N_a_ binding to

blocking anti-CD137 mAb (mAb values are constant)

NK_0_ death

TNF-α induced

apoptosis of APC

Activated NK with CD137L signaling (N_s_)

$$N_{s}(0)=0$$

NK_s_ proliferation

$\begin{matrix} \frac{dN_{s}}{dt}=\text{κ}_{\text{Na}}\cdot r\kappa\cdot N_{s}+\text{k}_{\text{(NxN)}}\cdot\text{rRLN}_{\text{a}}\cdot(\text{rRLN}_{\text{a}}\cdot2N_{a}+N_{Ab}+N_{s}+N_{s\_Ab})\cdot N_{a}+ \\ \text{k}_{\text{(AxN)}}\cdot\text{rRLN}_{\text{a}}\cdot(A_{a}+A_{s}+A_{Ab}+A_{s\_Ab})\cdot N_{a}-\text{μ}_{\text{Na}}\cdot N_{s}-\text{μαN}\cdot\left(\frac{\alpha}{\alpha+\text{K}_{\text{α}}\text{μA}}\right)\cdot N_{s}-\text{k}_{\text{(NxAb)}}\cdot Ab\cdot N_{s} \end{matrix}$ (S8)

N_s_ death

N_s_ binding to blocking anti-CD137 mAb

TNF-α induced

apoptosis of APC

Activated NK with the receptor bound to blocking anti-CD137 mAb (N_Ab_)

$$N_{Ab}(0)=0$$

TNF-α induced

apoptosis of APC

NK_Ab_ death

NK_Ab_ proliferation

**$\frac{dN_{Ab}}{dt}=\kappa_{Na}\cdot r\kappa\cdot N_{Ab}+\text{k}_{\text{(NxAb)}}\cdot Ab\cdot N_{s}-\text{μN}\cdot N_{Ab}-\text{μ}_{\text{Na}}\cdot\left(\frac{\alpha}{\alpha+\text{K}_{\text{α}}\text{μN}}\right)\cdot N_{Ab}$** (S9)

Activated NK, signalized by CD137::CD137L and with the receptor bound to blocking anti-CD137 mAb (N_s_Ab_)

$$N_{s\_Ab}(0)=0$$

TNF-α induced

apoptosis of APC

NK_s_Ab_ proliferation

NK_s_Ab_ death

$\frac{dN_{s\_Ab}}{dt}=\kappa_{Na}\cdot r\kappa\cdot N_{s}+\text{k}_{\text{(NxAb)}}\cdot Ab\cdot N_{s}-\text{μ}_{\text{Na}}\cdot N_{s\_Ab}-\text{μαN}\cdot\left(\frac{\alpha}{\alpha+\text{K}_{\alpha}\text{μN}}\right)\cdot N_{s\_Ab}$ (S10)

**TL cells dynamics**

Non-antigen-specific T cells (T_ns_)

$$T_{ns}(0)=\text{rT}\cdot\text{(1.0-rTsp)}$$

Tns death

Tns proliferation

$\frac{dT_{ns}}{dt}=-\text{μ}_{\text{T}_{0}}\cdot T_{ns}+\text{κT}_{o}\cdot\text{rκ}\cdot T_{ns}$ (S11)

Naïve specific T cells (T_0_)

T differentiation (induced by activated APC)

T_0_ proliferation

T_0_ death

$$T_{0}(0)=\text{rT}\cdot\text{rTsp}$$

$\frac{dT_{0}}{dt}=-\text{μ}_{\text{T}_{0}}\cdot T_{0}+\kappa T_{0}\cdot r\kappa\cdot T_{0}-\text{k}_{\text{(AxT)}}\cdot(A_{a}+A_{s}+A_{s\_Ab}+A_{Ab})\cdot T_{0}$ (S12)

TNF-α induced

apoptosis of APC

T cells activated by APC (with CD137:CD137L signaling) (T_s_)

Ts death

$$T_{s}(0)=0$$

$\frac{dT_{s}}{dt}=\text{k}_{\text{(AxT)}}\cdot\left(\frac{\text{T}_{\text{Ab}}}{\text{T}_{\text{Ab}}+ Ab}\right)\cdot(A_{a}+A_{s}+A_{s\_Ab}+A_{Ab})\cdot T_{0}+\text{κT}_{\text{s}}\cdot r\kappa\cdot T_{s}-\text{μT}_{\text{s}}\cdot T_{s}-\text{μαT}\cdot\left(\frac{\alpha}{\alpha+\text{K}_{\alpha}\text{μT}}\right)\cdot T_{s}$ (S13)

Ts proliferation

T differentiation (induced by activated APC)

T cells activated by APC (without CD137:CD137L signaling) (T_bl_)

$$T_{bl}(0)=0$$

TNF-α induced

apoptosis of T cells

Effect of CD137 blockade in Ts death

$\frac{dT_{bl}}{dt}=\text{k}_{\text{(AxT)}}\cdot\left(\frac{Ab}{T_{Ab}+ Ab}\right)\cdot T_{0}\cdot(A_{a}+A_{s}+A_{S_{Ab}}+A_{Ab})+\text{κT}_{s}\cdot\text{κI}_{\text{T}}\cdot T_{bl}-\text{μ}_{\text{Ts}}\cdot\text{IμT}\cdot T_{bl}-\text{μαT}\cdot\left(\frac{\alpha}{\alpha+\text{K}_{\text{α}}\text{μT}}\right)$ (S14)

Effect of CD137 blockade in Ts proliferation

T differentiation (induced by activated APC) with CD137::CD137L interaction blocked

**Media dynamics.**

*IFN-γ levels*

(S15)´´´

(S15)

(S15)´

(S15)´´

*TNF-α levels*

(S16)´´´

(S16)

(S16)´´

(S16)´

Antigen concentration

$Ag(0)=0$ (Media treatment)

$Ag(0)=10ng/ml$ (*M.tb* treatment and blocking treatment)

$\frac{dAg}{dt}=-\text{ μ}_{\text{Ag}}\cdot Ag$ (S17)

**Other APC equations**

Total number of APC

$A_{tot}= A_{0}+ A_{a}+ A_{Ab}+ A_{s}+ A_{s\_Ab}$ (R1)

Percentage of APC producing IFN-γ and TNF-α

**$r\gamma A= \frac{\text{rγA}_{0}\cdot A_{0}\text{+rγA}_{\text{a}}\cdot(A_{a}+A_{Ab}+ A_{s}+A_{s\_Ab})}{A_{tot}} \cdot100$** (R2)

**$r\alpha_{A}=\frac{ \text{rαA}_{0}\cdot A_{0}+A_{a}+A_{Ab}+ A_{s}+A_{s\_Ab}}{A_{tot}} \cdot100$** (R3)

Percentage of APC expressing receptor and ligand

$rRLA=\frac{ \text{rRLA}_{0}\cdot A_{0}+A_{a}+A_{Ab}+A_{s}+A_{s\_Ab}}{A_{tot}} \cdot100$ (R4)

**Other NK equations**

Total number of NK

$N_{tot}= N_{0}+ N_{a}+ N_{s}+ N_{s\_Ab}+ N_{Ab}$ (R5)

Percentage of NK producing IFN-γ and TNF-α

$r\gamma_{N}=\frac{\text{ rγN}_{0}\cdot N_{0}+N_{a}+ N_{s}+ N_{s\_Ab}+ N_{Ab}}{N_{tot}} \cdot100$ (R6)

**$r\alpha_{N}=\frac{ r\alpha N_{0}\cdot N_{0}+r\alpha N_{a}\cdot N_{a} (N_{a}+ N_{s}+ N_{s\_Ab}+ N_{Ab})}{N_{tot}}\cdot100$** (R7)

Percentage of NK expressing receptor and ligand

$rRLN=\frac{ \text{rRLN}_{0}\cdot N_{0}+ \text{rRLN}_{\text{a}}\cdot N_{a}+ N_{s}+N_{s\_Ab}+N_{Ab}}{N_{tot}}\cdot100$ (R8)

**Other TL equations**

Total number of TL

$T_{tot}=T_{ns}+ T_{0}+ T_{s}+ T_{bl}$ (R9)

Percentage of TL producing IFN-γ and TNF-α

$r\gamma_{T}=\frac{\text{rγT}_{0}\cdot(T_{ns} +T_{0})+\text{rγT}_{\text{S}}\cdot(T_{s}+T_{bl})}{T_{tot}} \cdot100$ (R11)

$r\alpha=\frac{ \text{rαT}_{0}\cdot(T_{ns}+T_{0})+\text{rαT}_{\text{s}}\cdot(T_{s}+T_{bl})}{T_{tot}} \cdot100$ (R12)

Percentage of TL expressing receptor

$rRT=\frac{ \text{rRT}_{0}\cdot T_{ns}+ \text{rRT}_{0}\cdot T_{0}+ T_{s}+T_{bl}}{T_{tot}} \cdot100$ (R10)

Percentage of TL undergoing apoptosis/necrosis

$TL_{apop }=\frac{100}{T_{tot}(t)}\int_{t-tA}^{t} \left(\text{μT}_{0}\cdot T_{ns}(\tau)+\text{ μT}_{0}\cdot T_{0}(\tau)+ \text{μT}_{\text{s} }\cdot T_{s}(\tau)+ \text{μαT}\cdot\left(\frac{\alpha(\tau)}{\alpha(\tau)+\text{K}_{\text{α}}\text{μT}}\right) \cdot\left(T_{s}(\tau)+T_{bl}(\tau)\right)+\text{μT}_{\text{s}}\cdot\text{μIT}\cdot T_{bl}(\tau)\right)d\tau$ (R13)

**Other Media equations.**

Total number of cells

$nC= A_{tot}+N_{tot}+T_{tot}$ (R14)

Proliferation ratio

$r\kappa=\frac{\text{mCells}-nC}{\text{mCells}}$ (R15)

### [tritiated thymidine](http://www.google.com.ar/url?sa=t&rct=j&q=triate+thymidine&source=web&cd=1&ved=0CCEQFjAA&url=http%3A%2F%2Flinkinghub.elsevier.com%2Fretrieve%2Fpii%2F0027510769901146&ei=L0NFT4quL8KyiQL368m5Dg&usg=AFQjCNEmIeh8UrHSZP4nXVdW_d-oCzaI7Q&sig2=WPa8Q2RI2uaxH3ydcEOQFQ)incorporation

 (R16)

Time dependent variables are written in *italics*, parameters are written in normal type

Table S1. List of parameter names, descriptions, units and prior and posterior parameters distribution. Parameters distributions are presented in ranges.

| **Name** | **Reference** | **units** | **Description** |
| --- | --- | --- | --- |
| **rA** | [[1](#_ENREF_1)] | ------ | Ratio of APC in PBMC |
| **rN** | [[2](#_ENREF_2)] | ------ | Ratio of NK^bright^ in PBMC |
| **rT** | [[2](#_ENREF_2)] | ------ | Ratio of TL PBMC |
| **rTsp** | [[3](#_ENREF_3)] [[4](#_ENREF_4)] | ------ | Ratio of TLspecific to M.tb |
| **γA_0_** | Estimated | ng A_0_^-1^ h^-1^ | IFNγ production by A_0_ |
| **γA_a_** | Estimated | ng A_a_^-1^ h^-1^ | IFNγ production by A_a_ |
| **γIA** | [[5](#_ENREF_5),[6](#_ENREF_6)] | ------ | Induction factor of IFNγ production by CD137::CD137L of APC |
| **αA_0_** | [[7](#_ENREF_7)] | ng A_a_^-1^ h^-1^ | TNFα production by A_0_ |
| **αA_a_** | [[7](#_ENREF_7)] | ng A_a_^-1^ h^-1^ | TNFα production by A_a_ |
| **αIA** | [[5](#_ENREF_5),[6](#_ENREF_6)] | ------ | Induction of TNFα production by CD137::CD137L of APC |
| **μA_0_** | [[7](#_ENREF_7)] | h^-1^ | Death rate for A_0_ |
| **μA_a_** | [[7](#_ENREF_7)] | h^-1^ | Death rate for A_a_ |
| **μIA** | [[8](#_ENREF_8)] [[9](#_ENREF_9)] | ------ | Induction of death by CD137::CD137L of APC |
| **μαA** | [[10](#_ENREF_10)] | h^-1^ | Maximum rate of TNF-α dependent apoptosis of APC |
| **K_α_μA** | [[7](#_ENREF_7)] | ng | Half-sat for TNF-dependent apoptosis of APC |
| **κA** | [[9](#_ENREF_9),[11](#_ENREF_11)] | h^-1^ | Proliferation rate of A_s_ |
| **rγA_0_** | Estimated | ------ | A_0_IFNγ^+^/A_0_ ratio |
| **rγA_a_** | Estimated | ------ | A_a_IFNγ^+^/A_a_ ratio |
| **rαA_0_** | Estimated | ------ | A_a_TNFα^+^/A_a_ ratio |
| **rRLA_0_** | Estimated | ------ | A_0_ CD137^+^ CD137L^+^ / A_0_ ratio |
| **k_(AxA)_** | [[7](#_ENREF_7)] | cell^-1^ h^-1^ | APC-APC interaction rate |
| **k_(AxN)_** | Estimated | cell^-1^ h^-1^ | APC-NK interaction rate |
| **k_(AxT)_** | [[7](#_ENREF_7)] | cell^-1^ h^-1^ | APC-LT interaction rate |
| **k_(AxAg)_** | [[7](#_ENREF_7)] | μg^-1^ h^-1^ | APC activation rate |
| **k_(AxAg)αγ_** | [[7](#_ENREF_7)] | μg^-1^ h^-1^ | APC-APC interaction rate (inflammatory context) |
| **k_(AxAb)_** | Estimated | μg^-1^ h^-1^ | Ab binding rate for APC |
| **K_γ(A0,Aa)_** | Estimated | μg^-1^ | Half-sat of IFN-γ on APC activation |
| **K_α(A0,Aa)_** | Estimated | μg^-1^ | Half-sat of TNF-α on APC activation |
| **γN_0_** | [[10](#_ENREF_10)] | ng^-1^ h^-1^ | IFNγ production by N_0_ |
| **γN_a_** | [[10](#_ENREF_10)] | ng^-1^ h^-1^ | IFNγ production by N_a_ |
| **γIN** | [[12](#_ENREF_12)] |  | Induction of IFNγ production by CD137 of NK |
| **αN_0_** | Estimated | μg^-1^ h^-1^ | TNFα production by N_0_ |
| **αN_a_** | [[13](#_ENREF_13)] | μg^-1^ h^-1^ | TNFα production by N_a_ |
| **αIN** | Estimated | ------ | Induction of TNFα production by CD137 of NK |
| **μN_0_** | [[14](#_ENREF_14)] | h^-1^ | Death rate for N_0_ |
| **μN_a_** | [[14](#_ENREF_14)] | h^-1^ | Death rate for N_a_ |
| **μ_α_N** | Estimated | h^-1^ | Maximum rate of TNF-α dependent apoptosis of NK |
| **K_α_μN** | Estimated | ng | TNF half-sat for TNF-α dependent apoptosis of NK |
| **κN_0_** | [[14](#_ENREF_14)] | h^-1^ | N_0_ Maximum proliferation rate |
| **κN_a_** | [[14](#_ENREF_14)] | h^-1^ | N_a_ Maximum proliferation rate |
| **rγN_0_** | [[15](#_ENREF_15)] | ------ | N_a_ IFN-γ^+^/N_a_ ratio |
| **rαN_0_** | [[16](#_ENREF_16)] | ------ | N_0_ TNF-α^+^/N_0_ ratio |
| **rαN_a_** | Estimated | ------ | N_a_ TNF-α^+^/N_a_ ratio |
| **rRLN_0_** | [[12](#_ENREF_12)] | ------ | N_0_ CD137^+^ CD137L^+^/N_0_ ratio |
| **rRLN_a_** | [[12](#_ENREF_12)] | ------ | N_a_ CD137^+^ CD137L^+^/N_a_ ratio |
| **k_(NxN)_** | Estimated | cell^-1^ h^-1^ | NK-NK interaction rate |
| **k_(NxAb)_** | Estimated | μg^-1^ h^-1^ | Ab binding rate for NK |
| **k_(N0,Na)A_** | Estimated | μg^-1^ cell^-1^ | Maximum NK activation rate, dependent on APC and indirect IL-12 |
| **K_A(N0,Na)_** | Estimated | μg cell | APC half-sat on enhancement of NK activation |
| **Kγ_(N0,Na)_** | Estimated | ng | Half-sat of IFN-γ on NK activation |
| **Kα_(N0,Na)_** | Estimated | ng | Half-sat of TNF-α on NK activation |
| **γT_0_** | [[17](#_ENREF_17)] | ng^-1^ h^-1^ | IFN-γ production rate by LT_0_ |
| **γT_s_** | [[10](#_ENREF_10)] | ng^-1^ h^-1^ | IFN-γ production rate by LT_s_ |
| **γIT** | [[18](#_ENREF_18)] [[19](#_ENREF_19)] | ------ | Induction of IFN-γ production by CD137 |
| **αT_0_** | Estimated | ng^-1^ h^-1^ | TNF-α production rate by T_0_ |
| **αT_s_** | [[10](#_ENREF_10)] [[20](#_ENREF_20)] [[21](#_ENREF_21)] | ng^-1^ h^-1^ | TNF-α production rate by T_s_ |
| **αIT** | [[19](#_ENREF_19)] | ------ | Induction of TNF-α production by CD137 |
| **μT_0_** | [[14](#_ENREF_14)] [[7](#_ENREF_7)] | h^-1^ | Apoptosis rate of T_0_ |
| **μT_s_** | [[7](#_ENREF_7)] | h^-1^ | Apoptosis rate of T_s_ |
| **μIT** | [[22](#_ENREF_22)] | ------ | Induction of apoptosis rate by CD137 |
| **μαT** | [[7](#_ENREF_7)] | h^-1^ | Maximum rate of TNF-dependent apoptosis of TL |
| **KαμT** | [[7](#_ENREF_7)] | ng | TNF half-sat for TNF-dependent apoptosis of TL |
| **κT_0_** | [[14](#_ENREF_14)] | h^-1^ | Proliferation rate of T_0_ |
| **κT_s_** | [[7](#_ENREF_7)] | h^-1^ | Proliferation rate of T_s_ |
| **κIT** | [[22](#_ENREF_22)] | ------ | Induction of proliferation rate by CD137 |
| **rγT_0_** | [[23](#_ENREF_23)] | ------ | T_0_ IFN-γ^+^/T_0_ ratio |
| **rγT_s_** | UD* | ------ | T_s_ IFN-γ^+^/T_s_ ratio |
| **rαT_0_** | Estimated | ------ | T_0_ TNF-α^+^/T_0_ ratio |
| **rαT_s_** | UD* | ------ | T_s_ TNF-α^+^/T_s_ ratio |
| **rRT_0_** | [[22](#_ENREF_22)] | ------ | T_0_CD137^+^/T_0_ ratio |
| **K_(TxAb)_** | Estimated | μg^-1^ h^-1^ | TL-mAb binding rate |
| **τAp** | [[24](#_ENREF_24)] | h | Duration of Apoptosis |
| **μα** |  | h^-1^ | Degradation rate of TNF-α |
| **μγ** | [[25](#_ENREF_25)] [[26](#_ENREF_26)] | h^-1^ | Degradation rate of IFN-γ |
| **μAg** |  | h^-1^ | Degradation/internalization rate of Ag |
| ***φ*Tym** | Estimated | cpm cell^-1^ | Scaling factor [H3]timidine-proliferation |
| **mCells** | Estimated | cell | Maximum number of cell supported by the media |

*UD=Unpublished data

References

1. Sleasman JW, Leon BH, Aleixo LF, Rojas M, Goodenow MM (1997) Immunomagnetic selection of purified monocyte and lymphocyte populations from peripheral blood mononuclear cells following cryopreservation. Clin Diagn Lab Immunol 4: 653-658.

2. Sotosek S, Sotosek Tokmadzic V, Mrakovcic-Sutic I, Tomas MI, Dominovic M, et al. (2011) Comparative study of frequency of different lymphocytes subpopulation in peripheral blood of patients with prostate cancer and benign prostatic hyperplasia. Wien Klin Wochenschr 123: 718-725.

3. Kaufmann SH, Vath U, Thole JE, Van Embden JD, Emmrich F (1987) Enumeration of T cells reactive with Mycobacterium tuberculosis organisms and specific for the recombinant mycobacterial 64-kDa protein. Eur J Immunol 17: 351-357.

4. Modlin RL, Melancon-Kaplan J, Young SM, Pirmez C, Kino H, et al. (1988) Learning from lesions: patterns of tissue inflammation in leprosy. Proc Natl Acad Sci U S A 85: 1213-1217.

5. Nguyen QT, Ju SA, Park SM, Lee SC, Yagita H, et al. (2009) Blockade of CD137 signaling counteracts polymicrobial sepsis induced by cecal ligation and puncture. Infect Immun.

6. Lee SC, Ju SA, Sung BH, Heo SK, Cho HR, et al. (2009) Stimulation of the molecule 4-1BB enhances host defense against Listeria monocytogenes infection in mice by inducing rapid infiltration and activation of neutrophils and monocytes. Infect Immun 77: 2168-2176.

7. Marino S, Myers A, Flynn JL, Kirschner DE (2010) TNF and IL-10 are major factors in modulation of the phagocytic cell environment in lung and lymph node in tuberculosis: a next-generation two-compartmental model. J Theor Biol 265: 586-598.

8. Langstein J, Becke FM, Sollner L, Krause G, Brockhoff G, et al. (2000) Comparative analysis of CD137 and LPS effects on monocyte activation, survival, and proliferation. Biochem Biophys Res Commun 273: 117-122.

9. Langstein J, Michel J, Schwarz H (1999) CD137 induces proliferation and endomitosis in monocytes. Blood 94: 3161-3168.

10. Sud D, Bigbee C, Flynn JL, Kirschner DE (2006) Contribution of CD8+ T cells to control of Mycobacterium tuberculosis infection. J Immunol 176: 4296-4314.

11. Langstein J, Schwarz H (1999) Identification of CD137 as a potent monocyte survival factor. J Leukoc Biol 65: 829-833.

12. Baessler T, Charton JE, Schmiedel BJ, Grunebach F, Krusch M, et al. (2010) CD137 ligand mediates opposite effects in human and mouse NK cells and impairs NK-cell reactivity against human acute myeloid leukemia cells. Blood 115: 3058-3069.

13. Millman AC, Salman M, Dayaram YK, Connell ND, Venketaraman V (2008) Natural killer cells, glutathione, cytokines, and innate immunity against Mycobacterium tuberculosis. J Interferon Cytokine Res 28: 153-165.

14. Lutz CT, Karapetyan A, Al-Attar A, Shelton BJ, Holt KJ, et al. (2011) Human NK cells proliferate and die in vivo more rapidly than T cells in healthy young and elderly adults. J Immunol 186: 4590-4598.

15. Alvarez IB, Pasquinelli V, Jurado JO, Abbate E, Musella RM, et al. (2010) Role played by the programmed death-1-programmed death ligand pathway during innate immunity against Mycobacterium tuberculosis. J Infect Dis 202: 524-532.

16. Ramana Rao PV, Rajasekaran S, Raja A (2010) Natural Killer Cell–Mediated Cytokine Response Among HIV-Positive South Indians With Pulmonary Tuberculosis. J Interferon Cytokine Res 30(1): 33-42.

17. Wigginton JE, Kirschner D (2001) A model to predict cell-mediated immune regulatory mechanisms during human infection with Mycobacterium tuberculosis. J Immunol 166: 1951-1967.

18. Shuford WW, Klussman K, Tritchler DD, Loo DT, Chalupny J, et al. (1997) 4-1BB costimulatory signals preferentially induce CD8+ T cell proliferation and lead to the amplification in vivo of cytotoxic T cell responses. J Exp Med 186: 47-55.

19. Zhang B, Maris CH, Foell J, Whitmire J, Niu L, et al. (2007) Immune suppression or enhancement by CD137 T cell costimulation during acute viral infection is time dependent. J Clin Invest 117: 3029-3041.

20. Rojas M, Olivier M, Gros P, Barrera LF, Garcia LF (1999) TNF-alpha and IL-10 modulate the induction of apoptosis by virulent Mycobacterium tuberculosis in murine macrophages. J Immunol 162: 6122-6131.

21. Li L, Sad S, Kagi D, Mosmann TR (1997) CD8Tc1 and Tc2 cells secrete distinct cytokine patterns in vitro and in vivo but induce similar inflammatory reactions. J Immunol 158: 4152-4161.

22. Wang C, Lin GH, McPherson AJ, Watts TH (2009) Immune regulation by 4-1BB and 4-1BBL: complexities and challenges. Immunol Rev 229: 192-215.

23. Jurado JO, Alvarez IB, Pasquinelli V, Martinez GJ, Quiroga MF, et al. (2008) Programmed death (PD)-1:PD-ligand 1/PD-ligand 2 pathway inhibits T cell effector functions during human tuberculosis. J Immunol 181: 116-125.

24. Potten C. S. , W. WJ (2004) Apoptosis: the life and death of cells: Cambridge University Prees.

25. Lortat-Jacob H, Baltzer F, Grimaud JA (1996) Heparin decreases the blood clearance of interferon-gamma and increases its activity by limiting the processing of its carboxyl-terminal sequence. J Biol Chem 271: 16139-16143.

26. Miyakawa N, Nishikawa M, Takahashi Y, Ando M, Misaka M, et al. (2011) Prolonged circulation half-life of interferon gamma activity by gene delivery of interferon gamma-serum albumin fusion protein in mice. J Pharm Sci 100: 2350-2357.
